# Socioeconomic determinants of use and choice of modern contraceptive methods in Ghana

**DOI:** 10.1186/s41182-022-00424-5

**Published:** 2022-05-17

**Authors:** Edward Nketiah-Amponsah, Samuel Ampaw, Priscilla Twumasi Baffour

**Affiliations:** 1 Present Address: Department of Economics, University of Ghana, Legon, Accra, Ghana; 2 School of Economics, University of Nottingham Ningbo China, Ningbo, People’s Republic of China

**Keywords:** Modern contraception, Family planning, Overpopulation, Sexually transmitted diseases, Fertility

## Abstract

**Background:**

The slow pace of fertility decline in Africa relative to other parts of the world has important implications for the region’s economic development. Modern contraceptive use is seen as important population control and family planning strategy by governments worldwide. This paper examines the socioeconomic determinants of modern contraceptive use and choice among Ghanaian men and women.

**Methods:**

We use the most recent and nationally representative Ghana Demographic and Health Survey conducted in 2014. The analysis is observational, with no causal implications. Bivariate and multivariate methods are used to analyse the data. We first use logistic regression to explore the correlates of modern contraceptive use among Ghanaian men and women. Second, we explore the socioeconomic factors influencing the choice of modern contraceptive methods among contraceptive users using multinomial logistic regression. We classify the modern methods of contraception into three groups: long-acting reversible contraceptives (LARC), short-acting contraceptives (SAC), and permanent contraceptives.

**Results:**

Marital status proves to be the most significant predictor for both men and women, with women in monogamous unions having a greater propensity to use modern methods of contraception (OR = 1.4, *p* < 0.00). We also find that different factors affect the use and choice of modern methods of contraception among men and women in Ghana. Muslim men had a higher likelihood than Catholics to choose the permanent (sterilisation) method (OR = 11.9, *p* < 0.05), while their female counterparts were 0.25 times less likely to choose sterilisation over SAC (*p* < 0.05). Moreover, women who ever tested for HIV had higher use of LAC than the SAC ((RRR = 1.6, *p* < 0.01). The modern contraceptive users (women) with at most basic education were more likely than those with tertiary education to choose LAC over SAC. Finally, rural women with health insurance were 0.75 times (*p* < 0.01) less likely to use modern methods of contraception.

**Conclusions:**

The paper reiterates the need to intensify and sustain public health education on the health benefits of using modern methods of contraception among the adult population. The paper suggests that including family planning services on Ghana’s national health insurance benefits package is commendable. It can promote modern contraceptive use and curtail unwarranted population growth.

**Supplementary Information:**

The online version contains supplementary material available at 10.1186/s41182-022-00424-5.

## Background

Access to safe and effective methods of contraceptives is seen as a fundamental human right. Modern contraceptive use remains a critical public health intervention, especially in less developed countries. HIV/AIDS, unintended pregnancies, and maternal mortality remain high in these countries [1]. Against this backdrop, target 3.7 of the Sustainable Development Goal (SDG) 3 seeks to achieve universal access to sexual and reproductive healthcare services, of which the use of modern contraceptives is paramount [2].

Globally, it is estimated that 76% of the 1.1 billion women who need family planning used modern contraceptives in 2019. Moreover, an estimated 190 million women in their reproductive age worldwide who want to prevent pregnancy do not use any contraceptive method. Furthermore, approximately 214 million women in low-and-middle-income countries who wanted to avoid pregnancy had an unmet need for any method of contraception in 2019 [2]. In sub-Saharan Africa, about 51 million women in the fertility bracket had an unmet need for modern contraceptive methods [3]. Stephenson et al. [4] note that modern contraceptive use (oral pills, condoms, intrauterine devices, sterilisation, implants, and injectables) has traditionally been low in sub-Saharan Africa, but evidence point to an increase in usage in the past decade. In addition, there are disparities in modern contraceptive use across SSA countries. In 2021, the prevalence of modern contraceptive usage among women in SSA ranged from a high of 65.8% in Zimbabwe to a low of 6.9% in Somalia [5].

Ghana's modern contraceptive prevalence rate has remarkably improved over the years from its lowest value of 5.2% in 1988 to 27.2% in 2021[4]. In 2017, only 26% of married Ghanaian women aged 15–49 who wanted to avoid pregnancy used modern methods of contraception [6], in sharp contrast to that of the USA (66%) and China (81%).^1^ In Ghana, the literature indicates that modern contraceptive use is influenced by high levels of education, low levels of infant and child mortality, marital status, employment status, health facility visits, age, religion, and cultural factors *inter alia* [4, 7–16]. Although studies abound on contraceptive use in Ghana, no single study has comprehensively studied the use and choice of modern methods of contraception from gender (male–female) and spatial perspectives (rural–urban) to ascertain whether modern contraceptive use differs by gender and space. We uniquely classify the modern methods of contraception into long-acting reversible contraceptives (LARC), short-acting contraceptives (SAC), and permanent contraceptives. This classification helps determine the peculiar predictors of users who prefer short-to-medium term reversible contraceptives vis-a-vis those who opt for permanent contraceptives. Finally, the study utilizes the 2014 GDHS, which is the most recent of the nationally representative DHSs in Ghana. Knowing the demographic and socioeconomic attributes of users of LARC, SAC, and permanent contraceptives provides useful information for policymakers to align reproductive health policies to the preferences of users.

## Methods

### Empirical model and statistical analysis

The analysis is observational, and no causal reading of the associations can be made. We first investigate the correlates of modern contraceptive use using logistic regression. This analysis aims to understand the characteristics of both men and women who use any modern method of contraception. After that, we explore the socioeconomic factors influencing the choice of modern contraceptive methods among the contraceptive users. A limitation here is that there might be multiple uses, but users of various forms are only classified according to the most effective method, as does DHS in reporting the modern method of contraception. We classify the modern methods of contraception into long-acting reversible contraceptives (LARC), short-acting contraceptives (SAC), and permanent contraceptives (see detail description in Table 1). This classification is useful in ascertaining the peculiar predictors of users who prefer short-to-medium term reversible contraceptives vis-a-vis those who opt for permanent contraceptives.

**Table 1:** Description and measurements of variables used in the estimations

Variables	Description and measurement
Modern contraceptive use	Current method of contraception: 1 = modern method, 0 = no method or traditional method (such as rhythm/periodic abstinence, withdrawal, “folk” method like herbs). The users of the traditional methods accounted for just 6% of the nonusers of modern methods in both the men’s and women’s samples
Modern contraceptive choice	Choice of modern method of contraception: 1 = short-acting contraceptives (jelly/foam, diaphragm, pill, or condom), 2 = long-acting reversible contraceptives (implants, intrauterine device, or injection), 3 = permanent/other methods (sterilisation or others)
Age	Respondent’s age: in years
Age squared	Age squared
Health insured	Health insurance status: 1 = insured, 0 = uninsured
Marital status	Marital status: 0 = unmarried; 1 = in monogamous union; 2 = in polygamous union
HIV tested	HIV testing status: 1 = ever tested, otherwise 0
Early sex	Age at first sex (early sex): 1 = below 17 years, 0 = 17 years plus
Urban	Place of residence: 1 = urban, 0 = rural
Wealth quintile	Wealth status: 1 = poorest, 2 = poorer, 3 = average, 4 = rich, 5 = richest^+^
Education	Educational attainment: 0 = no formal education, 1 = basic education, 2 = secondary education, 3 = tertiary education ^+^
Religion	Religious affiliation: 0 = no religion^+^, 1 = Catholic, 2 = Charismatic, 3 = other Christianity, 4 = Islam, 5 = Traditional
Region	Administrative region: 1 = Western, 2 = Central, 3 = Greater Accra^+^, 4 = Volta, 5 = Eastern, 6 = Ashanti, 7 = Brong Ahafo, 8 = Northern, 9 = Upper East, 10 = Upper West

Sample weights and clusters applied in the mean and standard error estimations, (+) represents the reference category

Following Jones [17], we hypothesise modern contraceptive choice as a function of the attributes $\left({X}_{ij}\right)$ woman* i* perceives in the *j*th contraceptive strategy, and personal socioeconomic characteristics $\left({R}_{i}\right)$. We formalise this idea as follows:

1 $${U}_{ij}={U}_{ij}\left({X}_{ij},{R}_{i}\right)+{\varepsilon }_{ij}$$

A woman’s choice can be viewed as comparing three competing utilities: *U*_1_*, U*_2_*,* and *U*_3_*,*_._ The woman chooses the strategy that maximises her satisfaction. The *U*_S_ are the indirect utilities the *i*th woman associates with the LARCs (1), SACs (2), and permanent contraceptives (3), respectively. Assuming a linear function, Eq. 1 can be reformulated as follows:

2 $${U}_{ij}={\propto }_{j}+{X}_{ij}{\beta }_{j}+{R}_{i}{\gamma }_{j}+{\varepsilon }_{ij}$$

Suppose an individual woman’s motivation is to maximise utility; she will choose the contraceptive strategy with the highest expected net benefit. The same principle applies to the choice of contraceptives among men.

Per our classification of the modern methods of contraception, the contraceptive user (consumer) faces three competing methods. Using a multinomial logit or probit to model the determinants of modern contraceptive choice might, therefore, produce consistent estimates. This classification is motivated by our objective to satisfy the assumption of independence of irrelevant alternatives (IIA), which is key in the estimation of multinomial logit. For analytic ease, we assume a logistic distribution for $({\varepsilon }_{i0}-{\varepsilon }_{ij})$. Thus, the appropriate model specification chosen for this study is the multinomial logit (MNL) which is consistent with the approach used by Jones [17].

Let the variable *Y* be a random variable indicating the choice made by an individual woman. Then an MNL model for $P\left(Y=j\right), j=\mathrm{1,2},3$ can be formulated as

3 $$P\left(Y=j\right)={e}^{X\beta +R\gamma \left(j\right)}/({e}^{X\beta +R\gamma \left(1\right)}+{e}^{X\beta +R\gamma \left(2\right)}+{e}^{X\beta +R\gamma \left(3\right)})$$

To estimate Eq. 3, $\beta ($2) is arbitrarily set to 0. Hence, the remaining coefficients $\beta$(1) and $\beta$(3) measure the change relative to the “reference category”. We use Stata 17.0 for all statistical analyses. In addition, we account for the survey design (sampling weights and clustering) using the multinomial logit command (*mlogit*) with *svy*.

### Data source and its limitations

Data from the 2014 Ghana Demographic and Health Survey (GDHS) were used for the study. The 2014 GDHS is the most recent of the nationally representative DHS implemented by the Ghana Statistical Service (GSS), Ghana Health Service (GHS), and ICF International. The survey followed a two-stage sampling procedure [18]. In the first stage, clusters constituting enumeration areas (EAs) were selected randomly. Subsequently, households were systematically sampled to be interviewed. Altogether, 11,835 households were interviewed, yielding a response rate of 98.5 per cent. A total of 9396 women aged 15–49 years and 4,388 men aged 15–59 years were successfully studied from the selected households [18]. These also yielded response rates of 97.3 per cent and 95.2 per cent, respectively.

The study uses data on 3852 men aged 15–59 years and 9,380 women aged 15–49 years to model the socioeconomic determinants of modern contraceptive use among men and women in Ghana. Some respondents were excluded from the analysis due to missing observations. The respective samples were further disaggregated on rural–urban basis. In modelling the determinants of choice of modern contraception, 819 men and 1731 women were utilised. These are the respondents who reported using modern contraception. This analysis does not disaggregate the samples on a rural–urban basis due to the smallness of the subgroups. For instance, only 5% of the men and 9% of the women used the permanent method of modern contraception.

### Variable description and measurement

Based on the extant literature, relevant socioeconomic characteristics of Ghanaian men and women were selected for the study. Table 1 describes and indicates the measurement of the dependent and independent variables used in the study. It is important to note that the inclusion of the explanatory variables is informed by both theory and the empirical literature [7, 10, 13]. For instance, marital status is a key predictor of modern methods of contraception. Notably, married couples use contraceptives to space childbirth and prevent STDs (in the case of condoms) and pregnancies (after reaching their desired number of children) [13]. In addition, religious affiliation may explain the uptake of modern methods of contraception, given that some religious denominations, including the Catholic faith, discourage the use of modern contraception [7, 13]. Moreover, income (wealth) indicates the ability to afford healthcare services and family planning methods, including modern methods of contraception.

**Fig. 1:**
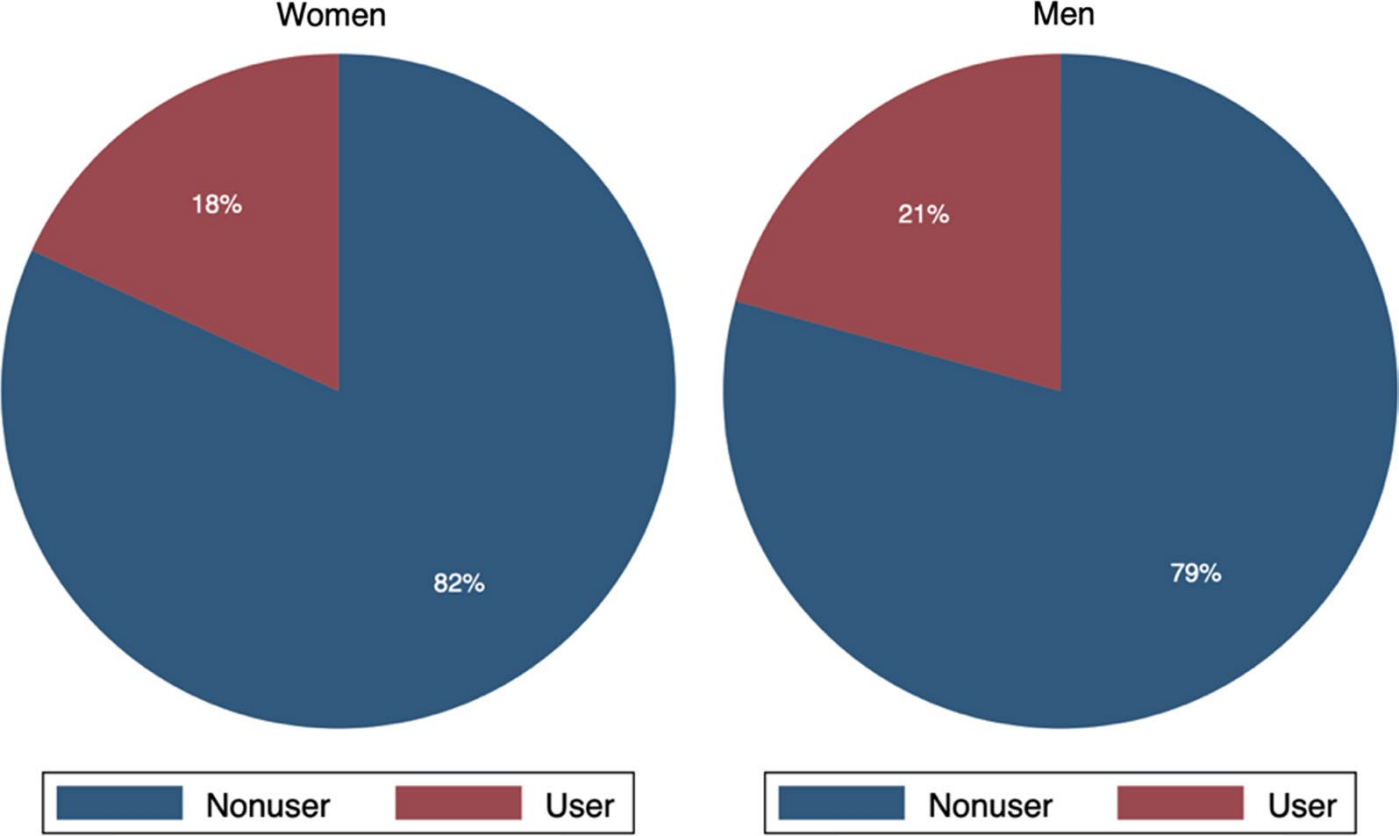
Modern contraceptive use by gender

Figure 1 shows that more men than women used modern methods of contraception. The difference was just 3 percentage points; however, sampling weights were applied, making the statistics nationally representative. The gender differences in modern methods of contraception are consistent with the literature. Biddlecom and Fapohunda [19] assert that the differences in the use of modern methods of contraception by men and women could be attributable to the ‘wedge’ between reported versus actual usage. Men are more likely to declare methods of contraception, especially when they play an active role in the relationship. At the same time, women fail to report usage of specific forms of contraception, especially when their partners disapprove of such methods. The covert use of contraception among women is more pervasive when there is ineffective spousal communication [19].

Ezeh and Mboup [20] examined the gender differences in contraceptive use in five developing countries (Central African Republic, Ghana, Haiti, Kenya, and Zambia) using the DHS data. They concluded that men are more likely to overreport contraceptive use, while women tend to underreport the same, owing to covert use (i.e., when women use a method of contraception without their husbands’ or partners’ knowledge). The gender differences could also be explained by differences in understanding the rationale for contraceptive use and the interpretation of the questionnaires about modern contraception, especially where literacy is low.

**Fig. 2:**
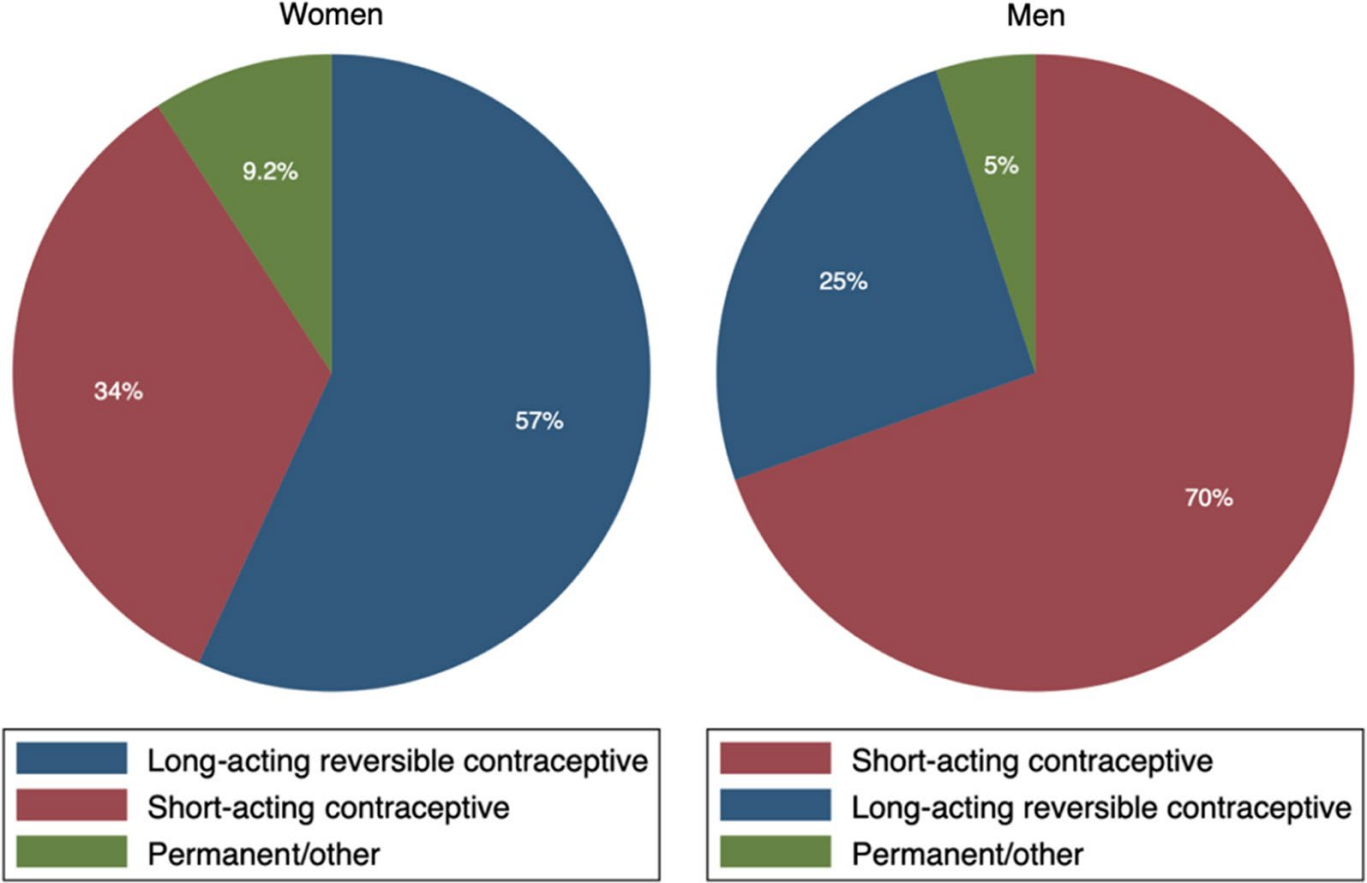
Choice of modern method of contraception by gender

Furthermore, Fig. 2 reveals that most men who used modern methods of contraception resorted to short-acting contraceptives. The male condom was the dominant option in this category. Men preferred condoms because of their potency in preventing pregnancy and sexually transmitted diseases [18, 19]. On the contrary, the female modern contraceptive users preferred the long-acting reversible methods to the other options. Most women used the injection method, followed by implant and IUD. The injection method is chosen probably because of its relative efficacy or reliability over pills, condoms, etc. In addition, for women, pills use was the commonest among the short-acting contraceptives, followed by the condom. The least preferred alternative among men and women was the permanent method (sterilisation). We, therefore, combine this method and those classified as “other modern methods” in the data to aid our empirical analyses.

## Results

### Bivariate analyses

Table 2 presents the results from a bivariate analysis of the selected socioeconomic characteristics of the men and women by use of modern contraception. HIV testing status, engagement in early sex, marital status, wealth status, educational attainment, religious affiliation, and administrative region of residence emerged statistically significant among both genders. In general, the modern contraceptive users had the following characteristics: higher HIV testing rate, higher incidence of early sex, lower likelihood of residing in the Northern Region, and a lower chance of association with the African traditional and Islamic Religions. The finding for region of residence is particularly intriguing, since the Northern Region is among Ghana's top three poorest regions. Its residents are predominantly Muslims and are more likely to use traditional or no method of contraception partly due to the preferences for large families, especially among the men.

**Table 2:** Bivariate analyses of socioeconomics characteristics by modern contraceptive use

Variables	Men				Women			
	Total (*N* = 4385)	Nonuser (*N* = 3495)	User (*N* = 890)	*p *value	Total (*N* = 9353)	Nonuser (*N* = 7628)	User (*N* = 1725)	*p* value
Age	32	32	33	0.27	30	30	31	**0.00**
Health insured	0.49	0.49	0.49	0.92	0.63	0.63	0.61	0.30
HIV tested	0.23	0.21	0.30	**0.00**	0.48	0.46	0.61	**0.00**
Early sex	0.18	0.17	0.23	**0.01**	0.32	0.30	0.42	**0.00**
Urban	0.53	0.53	0.53	0.85	0.54	0.55	0.47	**0.00**
*Marital status*								
Unmarried	0.48	0.47	0.50	0.42	0.44	0.46	0.31	**0.00**
Monogamous union	0.48	0.48	0.48	0.80	0.48	0.45	0.60	**0.00**
Polygamous union	0.04	0.04	0.03	**0.01**	0.09	0.09	0.09	0.83
*Wealth quintile*								
Poorest	0.17	0.18	0.12	**0.00**	0.16	0.16	0.16	0.73
Poorer	0.18	0.18	0.16	0.43	0.18	0.17	0.20	0.06
Middle	0.19	0.18	0.22	0.08	0.21	0.20	0.24	**0.00**
Richer	0.22	0.22	0.22	0.90	0.23	0.23	0.20	0.06
Richest	0.25	0.24	0.29	**0.04**	0.23	0.24	0.20	**0.03**
*Educational attainment*								
None	0.11	0.12	0.06	**0.00**	0.19	0.19	0.18	0.30
Basic	0.13	0.14	0.13	0.48	0.18	0.17	0.21	**0.01**
Secondary	0.64	0.65	0.63	0.47	0.57	0.57	0.55	0.15
Tertiary	0.12	0.10	0.18	**0.00**	0.06	0.06	0.06	0.90
*Religious affiliation*								
Catholic	0.11	0.10	0.13	0.09	0.10	0.10	0.11	0.34
Charismatic	0.31	0.30	0.31	0.87	0.41	0.41	0.43	0.33
Other Christian	0.32	0.31	0.34	0.19	0.29	0.29	0.31	0.28
Islam	0.18	0.18	0.14	**0.01**	0.15	0.16	0.12	**0.00**
Traditional	0.04	0.04	0.02	**0.02**	0.02	0.02	0.01	**0.03**
None	0.06	0.06	0.06	0.92	0.03	0.03	0.03	0.19
*Administrative region*								
Western	0.12	0.12	0.11	0.58	0.11	0.11	0.12	0.25
Central	0.10	0.08	0.17	**0.01**	0.10	0.09	0.13	0.06
Greater Accra	0.22	0.21	0.26	0.07	0.20	0.21	0.17	0.07
Volta	0.07	0.08	0.05	**0.02**	0.08	0.07	0.10	**0.04**
Eastern	0.10	0.10	0.09	0.29	0.09	0.09	0.09	0.92
Ashanti	0.18	0.19	0.15	0.06	0.19	0.20	0.16	0.10
Brong Ahafo	0.08	0.08	0.08	0.86	0.08	0.08	0.11	**0.01**
Northern	0.08	0.09	0.04	**0.00**	0.08	0.09	0.04	**0.00**
Upper East	0.04	0.04	0.04	0.54	0.04	0.04	0.04	0.95
Upper West	0.02	0.02	0.02	0.32	0.02	0.02	0.02	0.52

Sampling weights applied to make estimates nationally representative; means and *p* values of adjusted Wald test for association reported (*H*_*o*_: *no relationship*), bold indicate statistically significant values at 95% confidence interval (*p* < 0.05)

Moreover, fewer men in polygamous unions used modern methods of contraception. Unmarried women were less likely to use modern methods of contraception. In contrast, more women in monogamous marriages used contraception. Whereas men in the highest wealth quintile (richest) had a higher modern contraceptive usage, their female counterparts who used the modern methods were less than the nonusers. However, women in the average wealth quintile who used modern methods of contraception were more than the nonusers. In addition, modern contraceptive use was lower among men with no formal education and higher among those with tertiary education. Education was relevant among the women with basic education only. Notably, the women with basic education were more likely to use modern methods of contraception.

In addition, the data suggests that age and residence are unique predictors of modern contraceptive use among women. Urban women who did not use modern methods of contraception were more than the users. In addition, on average, the users were a year older than the nonusers.

Table 3 presents the results for correlates of choice of modern method of contraception. The table shows that the choice of modern method of contraception is highly associated with age, marital status, educational attainment, and administrative region of residence. These variables are statistically significant among both men and women.

**Table 3:** Bivariate analyses of socioeconomics characteristics by choice of modern method of contraception

Variables	Men				Women			
	LARC	SAC	Permanent/other	*p* value	LARC	SAC	Permanent/other	*p*-value
	(*N* = 229)	(*N* = 624)	(*N* = 37)		(*N* = 1026)	(*N* = 568)	(*N* = 131)	
Age	38	31	40	**0.00**	31	29	38	**0.00**
Health insured	0.52	0.48	0.56	0.50	0.63	0.58	0.64	0.25
HIV tested	0.32	0.29	0.38	0.56	0.64	0.55	0.60	**0.01**
Engaged in early sex	0.19	0.24	0.22	0.45	0.44	0.40	0.40	0.62
Urban	0.44	0.57	0.47	0.08	0.42	0.54	0.56	**0.01**
*Marital status*								
Unmarried	0.07	0.67	0.25	**0.00**	0.24	0.44	0.27	**0.00**
Monogamous union	0.86	0.31	0.75	**0.00**	0.66	0.50	0.63	**0.00**
Polygamous union	0.07	0.01	0.00	**0.00**	0.10	0.06	0.10	**0.01**
*Wealth quintile*								
Poorest	0.15	0.11	0.06	0.06	0.19	0.12	0.07	**0.00**
Poorer	0.21	0.15	0.16	0.16	0.22	0.16	0.21	**0.01**
Middle	0.23	0.21	0.30	0.44	0.25	0.23	0.20	0.52
Richer	0.18	0.24	0.18	0.43	0.19	0.24	0.18	0.16
Richest	0.23	0.31	0.31	0.41	0.15	0.25	0.34	**0.01**
*Educational attainment*								
None	0.12	0.04	0.04	**0.00**	0.21	0.12	0.20	**0.00**
Basic	0.16	0.12	0.05	**0.03**	0.24	0.16	0.20	**0.00**
Secondary	0.54	0.66	0.70	**0.03**	0.50	0.63	0.55	**0.00**
Tertiary	0.18	0.18	0.21	0.94	0.05	0.10	0.05	0.07
*Religious affiliation*								
Catholic	0.13	0.14	0.03	**0.00**	0.11	0.11	0.06	0.17
Charismatic	0.31	0.31	0.34	0.92	0.44	0.39	0.48	0.14
Other Christian	0.34	0.32	0.51	0.18	0.29	0.34	0.28	0.16
Islam	0.13	0.15	0.08	0.28	0.11	0.12	0.10	0.82
Traditional	0.03	0.02	0.02	0.50	0.01	0.01	0.01	0.95
None	0.06	0.06	0.01	**0.00**	0.03	0.02	0.07	0.29
*Administrative region*								
Western	0.05	0.13	0.13	**0.01**	0.11	0.15	0.12	0.45
Central	0.22	0.15	0.15	0.38	0.12	0.12	0.24	0.18
Greater Accra	0.27	0.26	0.20	0.70	0.15	0.20	0.16	0.29
Volta	0.05	0.06	0.02	0.28	0.10	0.12	0.04	**0.00**
Eastern	0.05	0.10	0.11	0.15	0.10	0.08	0.12	0.29
Ashanti	0.14	0.14	0.33	0.11	0.16	0.16	0.23	0.34
Brong Ahafo	0.10	0.07	0.03	0.15	0.12	0.10	0.07	0.16
Northern	0.02	0.05	0.02	0.10	0.05	0.03	0.01	**0.02**
Upper East	0.08	0.03	0.01	**0.00**	0.05	0.02	0.00	**0.00**
Upper West	0.02	0.02	0.00	**0.00**	0.03	0.02	0.01	**0.01**

Sampling weights applied to make estimates nationally representative; means and *p* values of adjusted Wald test for association reported (*H*_*o*_: *no relationship*), bold indicate statistically significant values at 95% confidence interval (*p* < 0.05)

On average, the users of the permanent method (sterilisation) were more advanced in age, followed by the LARC and SAC users, respectively. In addition, the largest proportion of the LARC and sterilisation users was in monogamous unions. Besides, while most male SAC users were unmarried, people in monogamous marriages dominated their female counterparts. Wealth status, HIV testing status, and place of residence proved statistically significant in the female sample only. Likewise, religion emerged significant in the male sample only.

### Multivariate analyses

#### Logistic regression: modern contraceptive use

Table 4 presents the odds ratios from the logistic regression model. These results show the determinants of modern contraceptive use among Ghanaian men and women. Notably, column (2) reports the results with interaction terms. We interact the significant variables in column (1) with rural–urban residence to explore whether these variables differ by place of residence. (Please see Tables D and E in Additional file 1 for separate estimates for the rural and urban samples).

**Table 4:** Determinants of modern contraceptive use

Explanatory variables	Men (*N* = 4385)		Women (*N* = 9353)	
	(1)	(2)	(1)	(2)
	OR [95% CI]	OR [95% CI]	OR [95% CI]	OR [95% CI]
Age	1.32^***^	1.32^***^	1.30^***^	1.30^***^
	**[1.23**–**1.41]**	**[1.23**–**1.41]**	**[1.23**–**1.39]**	**[1.22**–**1.39]**
Age squared	0.96^***^	0.96^***^	0.96^***^	0.96^***^
	**[0.96**–**0.97]**	**[0.96**–**0.97]**	**[0.95**–**0.97]**	**[0.95**–**0.97]**
*Health insurance status*				
Uninsured	1	1	1	1
Insured	1.09	1.07	0.84^*^	0.77^**^
	[0.89–1.35]	[0.87–1.32]	**[0.73**–**0.97]**	**[0.64**–**0.92]**
*Marital status*				
Unmarried	1	1	1	1
In monogamous union	0.50^***^	0.65^**^	1.44^***^	1.52^***^
	**[0.38**–**0.66]**	**[0.47**–**0.90]**	**[1.20**–**1.73]**	**[1.20**–**1.92]**
In polygamous union	0.48^**^	0.56	1.24	1.38^*^
	**[0.29**–**0.79]**	[0.30–1.07]	[0.97–1.58]	**[1.03**–**1.85]**
*HIV testing status*				
Not tested	1	1	1	1
Tested	1.19	1.20	1.42^***^	1.37^**^
	[0.92–1.54]	[0.93–1.56]	**[1.20**–**1.67]**	**[1.09**–**1.72]**
*Engaged in early sex (age* < *17 years)*				
No	1	1	1	1
Yes	1.47^**^	1.80^***^	1.64^***^	1.58^***^
	**[1.13**–**1.91]**	**[1.30**–**2.51]**	**[1.40**–**1.91]**	**[1.31**–**1.89]**
*Wealth quintile (Richest)*				
Richest	1	1	1	1
Poorest	0.81	0.79	1.38	1.38
	[0.46–1.43]	[0.45–1.39]	[0.96–1.97]	[0.96–1.98]
Poorer	0.97	0.92	1.20	1.20
	[0.60–1.57]	[0.57–1.47]	[0.83–1.73]	[0.83–1.74]
Middle	1.17	1.03	1.23	1.24
	[0.76–1.80]	[0.67–1.59]	[0.94–1.60]	[0.95–1.62]
Richer	0.96	0.87	1.01	1.01
	[0.66–1.38]	[0.59–1.26]	[0.81–1.26]	[0.81–1.27]
*Educational attainment*				
Tertiary	1	1	1	1
None	0.46^**^	0.26^***^	0.75	0.75
	**[0.28**–**0.77]**	**[0.13**–**0.50]**	[0.49–1.14]	[0.49–1.14]
Basic	0.74	0.42^**^	0.94	0.95
	[0.48–1.14]	**[0.24**–**0.74]**	[0.63–1.40]	[0.64–1.41]
Secondary	0.73	0.52^**^	0.91	0.92
	[0.53–1.02]	**[0.32**–**0.85]**	[0.62–1.33]	[0.63–1.34]
*Religious affiliation*				
Catholic	1	1	1	1
Charismatic	1.25	1.23	0.94	0.95
	[0.78–2.03]	[0.76–2.00]	[0.62–1.43]	[0.63–1.45]
Other Christian	0.96	0.95	0.90	0.91
	[0.64–1.46]	[0.62–1.45]	[0.61–1.33]	[0.61–1.34]
Islam	1.06	1.05	0.93	0.94
	[0.69–1.65]	[0.67–1.64]	[0.64–1.35]	[0.65–1.35]
Traditional	0.92	0.87	0.77	0.79
	[0.58–1.48]	[0.53–1.42]	[0.53–1.13]	[0.54–1.15]
None	0.89	0.86	0.54^*^	0.53^*^
	[0.50–1.56]	[0.49–1.51]	**[0.29**–**1.00]**	**[0.29**–**0.99]**
*Administrative region*				
Greater Accra	1	1	1	1
Western	0.72	0.70	1.23	1.22
	[0.47–1.10]	[0.46–1.07]	[0.88–1.72]	[0.87–1.70]
Central	1.78^**^	1.70^*^	1.39	1.38
	**[1.17**–**2.71]**	**[1.14**–**2.55]**	[1.00–1.94]	[0.99–1.93]
Volta	0.51^**^	0.50^**^	1.47^*^	1.47^*^
	**[0.32**–**0.84]**	**[0.31**–**0.82]**	**[1.02**–**2.13]**	**[1.01**–**2.12]**
Eastern	0.71	0.71	1.03	1.02
	[0.48–1.07]	[0.48–1.07]	[0.74–1.43]	[0.74–1.42]
Ashanti	0.63^*^	0.62^*^	0.93	0.93
	**[0.42**–**0.93]**	**[0.42**–**0.92]**	[0.68–1.29]	[0.67–1.28]
Brong Ahafo	0.88	0.87	1.47^*^	1.45^*^
	[0.56–1.38]	[0.56–1.37]	**[1.06**–**2.03]**	**[1.04**–**2.01]**
Northern	0.60	0.61	0.51^**^	0.51^**^
	[0.33–1.08]	[0.34–1.10]	**[0.34**–**0.77]**	**[0.34**–**0.77]**
Upper East	1.32	1.40	1.08	1.07
	[0.75–2.31]	[0.81–2.42]	[0.75–1.56]	[0.74–1.55]
Upper West	0.86	0.91	1.22	1.22
	[0.51–1.43]	[0.55–1.52]	[0.82–1.82]	[0.82–1.81]
*Place of residence*				
Rural	1	1	1	1
Urban	0.77	0.60	0.83	0.72
	[0.56–1.05]	[0.34–1.06]	[0.66–1.03]	[0.51–1.02]
Insured#urban				1.22
				[0.93–1.61]
Early sex#urban		0.69		1.10
		[0.41–1.16]		[0.81–1.48]
Tested#urban				1.09
				[0.77–1.53]
*Marital status#urban*				
In monogamous union#urban		0.62^*^		0.91
		**[0.39**–**0.98]**		[0.66–1.23]
In polygamous union#urban		0.67		0.75
		[0.25–1.85]		[0.46–1.20]
*Education#urban*				
None#urban		3.67^**^		
		**[1.48**–**9.12]**		
Basic#urban		2.89^**^		
		**[1.31**–**6.39]**		
Secondary#urban		1.65		
		[0.89–3.05]		

OR: odds ratio, results from logistic regression, survey design (sampling weights and clustering) applied, bold indicate statistically significant values at 95% confidence interval (CI), *p < 0.05, **p < 0.01, ***p < 0.001

**Fig. 3:**
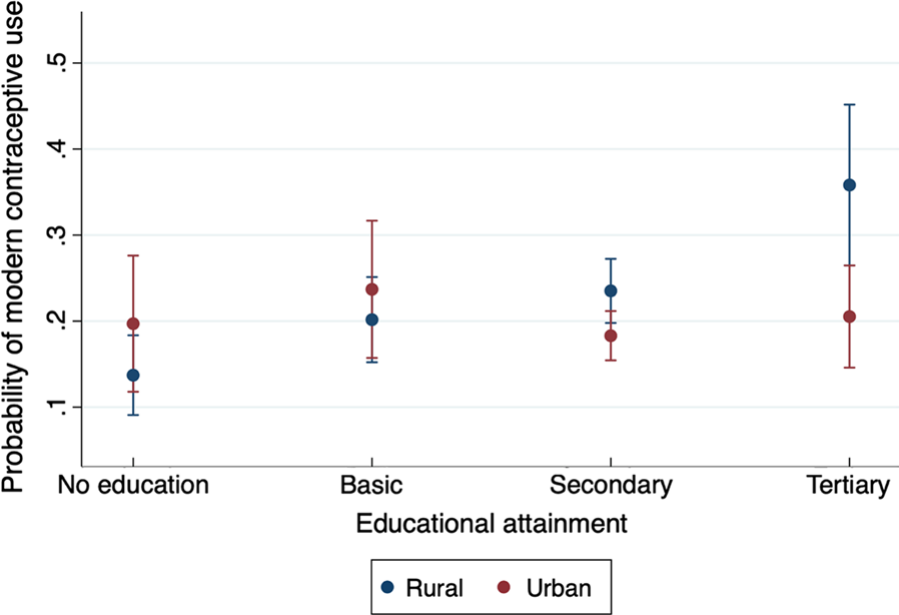
Predictive margins of interaction between educational attainment and urban–rural residence with 95% confidence interval

The joint tests of the interaction terms are statistically insignificant in most cases, except for education in the men’s data (see Fig. 3 for a graphical representation of the results). The overall statistical significance of the respective models confirms the robustness of the models in explaining the determinants of modern contraceptive use among Ghanaian men and women. (See Tables D and E in Additional file 1.) We focus on column 1 results, since the *F*-statistics of the models without the interaction terms are greater than those with interaction terms.

The determinants of modern contraceptive use vary substantially by gender but not much by rural–urban residence. Consistent with the bivariate analysis, marital status, early sex, and region are statistically significant among Ghanaian men and women. Age was also crucial among both genders, though statistically significant in the women sample only in the bivariate analysis.

Compared with unmarried men, those in monogamous and polygamous marriages were less likely to use modern methods of contraception (OR = 0.05, *p* < 0.001 and OR = 0.48, *p* < 0.01, respectively). In contrast, women in monogamous marriages had a higher likelihood of using the modern methods (OR = 1.4, *p* < 0.001) than their unmarried counterparts. Furthermore, we find an inverted U-shaped relationship between age and modern contraceptive use among both genders (age: OR = 1.3, *p* < 0.001; age squared: OR = 0.96, *p* < 0.01). The results additionally show that men (OR = 1.5, *p* < 0.01) and women (OR = 1.6, *p* < 0.001) who had sex before their seventeenth birthday were more likely to use modern methods of contraception. In addition, revealing is the finding that women in the Northern Region (OR = 0.51, *p* < 0.01) were less likely to use modern methods of contraception relative to those in the Greater Accra region.

Moreover, the data suggest that some of the determinants are gender specific. For instance, health insurance status (OR = 0.84, *p* < 0.05), HIV testing status (OR = 1.4, *p* < 0.001), and religion (no religion: OR = 0.54, *p* < 0.05) proved to be significant predictors for women only. Education was likewise significant among just men (no education: OR = 0.46, *p* < 0.01). Figure 3 further shows that urban men with at most basic education were more likely to use modern methods of contraception than their counterparts in rural areas. However, rural men with at least secondary education were more likely to use modern methods of contraception than their urban counterparts.

#### Multinomial logistic regression: modern contraceptive choice

Table 5 shows the relative-risk ratios from the multinomial logistic regression models for the male and female samples. The statistical significance of the *F*-statistics for the goodness of fit suggests that the models fit the data well. The short-acting contraceptive method was used as the base outcome in both samples. By default, Stata uses that category as the reference in the male sample estimation, perhaps because it had the highest frequency. We use the SACs as the reference category for two reasons. (1) To ensure that our findings are comparable across gender groups. (2) We are more interested in why people would choose the LARC and Permanent methods over the SAC. The SACs are the least effective modern methods of contraception (performance- and costwise). The generalised Hausman test for IIA fails to reject the null hypothesis that the difference in coefficients is not systematic. By implication, the IIA assumption, necessary for multinomial logistic regression, is not rejected.

**Table 5:** Determinants of modern contraceptive choice

Explanatory variables	Men (*N* = 890)		Women (*N* = 1725)	
	LARC	Permanent/other	LARC	Permanent/other
	RRR [95% CI]	RRR [95% CI]	RRR [95% CI]	RRR [95% CI]
Age	1.16	0.92	1.16^*^	1.03
	[0.93–1.45]	[0.69–1.23]	**[1.03**–**1.31]**	[0.75–1.41]
Age squared	0.98	1.02	0.977^*^	1.01
	[0.95–1.01]	[0.98–1.06]	**[0.959**–**0.996]**	[0.97–1.06]
*Health insurance status*				
Uninsured	1	1	1	1
Insured	1.01	1.04	1.20	1.75
	[0.63–1.61]	[0.45–2.41]	[0.91–1.60]	[0.95–3.21]
*Marital status*				
Unmarried	1	1	1	1
In monogamous union	21.59^***^	3.75^*^	1.86^***^	0.91
	**[8.17**–**57.06]**	**[1.08**–**13.02]**	**[1.36**–**2.54]**	[0.46–1.78]
In polygamous union	62.13^***^	0.00^***^	2.32^**^	1.03
	**[16.49**–**234.13]**	**[0.00**–**0.00]**	**[1.39**–**3.88]**	[0.31–3.38]
*HIV testing status*				
Not tested	1	1	1	1
Tested	1.08	1.15	1.55^**^	1.45
	[0.60–1.95]	[0.50–2.64]	**[1.15**–**2.08]**	[0.82–2.59]
*Engaged in early sex (age < 17 years)*				
No	1	1	1	1
Yes	0.80	0.76	1.04	1.25
	[0.44–1.47]	[0.23–2.57]	[0.70–1.55]	[0.75–2.10]
*Wealth quintile (Richest)*				
Richest	1	1	1	1
Poorest	1.28	0.60	1.96	0.40
	[0.32–5.18]	[0.05–6.77]	[0.90–4.25]	[0.11–1.46]
Poorer	2.04	0.85	2.19^*^	0.71
	[0.68–6.13]	[0.14–4.98]	**[1.19**–**4.01]**	[0.29–1.74]
Middle	2.32	1.82	1.92^*^	0.60
	[0.79–6.80]	[0.35–9.61]	**[1.15**–**3.20]**	[0.28–1.28]
Richer	1.41	1.00	1.29	0.50
	[0.49–4.06]	[0.26–3.80]	[0.78–2.14]	[0.23–1.08]
*Educational attainment*				
Tertiary	1	1	1	1
None	1.47	0.80	2.72^*^	3.19
	[0.49–4.39]	[0.12–5.27]	**[1.20**–**6.14]**	[0.99–10.29]
Basic	1.04	0.35	2.53^*^	1.97
	[0.41–2.67]	[0.06–1.94]	**[1.20**–**5.34]**	[0.53–7.23]
Secondary	0.93	0.80	1.56	1.94
	[0.43–2.05]	[0.29–2.23]	[0.73–3.32]	[0.77–4.91]
*Religious affiliation*				
Catholic	1	1	1	1
Charismatic	1.11	2.47	0.71	0.20
	[0.39–3.18]	[0.15–39.86]	[0.29–1.71]	[0.04–1.09]
Other Christian	1.32	10.96^*^	0.94	0.40
	[0.44–3.90]	**[1.16**–**103.43]**	[0.41–2.17]	[0.12–1.31]
Islam	1.02	11.94^*^	0.80	0.25^*^
	[0.37–2.81]	**[1.20**–**118.59]**	[0.33–1.92]	**[0.07**–**0.89]**
Traditional	0.60	6.28	0.51	0.24
	[0.19–1.92]	[0.48–81.54]	[0.20–1.30]	[0.05–1.28]
None	0.87	42.74^**^	0.55	0.50
	[0.21–3.51]	**[3.20**–**571.40]**	[0.16–1.86]	[0.08–2.90]
*Administrative region*				
Greater Accra	1	1	1	1
Western	0.25^*^	1.48	0.79	1.25
	**[0.08**–**0.76]**	[0.31–7.13]	[0.41–1.49]	[0.54–2.89]
Central	0.53	0.76	0.97	2.33
	[0.19–1.43]	[0.13–4.66]	[0.53–1.77]	[0.95–5.69]
Volta	0.43	0.35	0.63	0.34
	[0.13–1.39]	[0.02–6.34]	[0.34–1.16]	[0.11–1.05]
Eastern	0.30	1.33	1.09	1.39
	[0.08–1.10]	[0.26–6.78]	[0.61–1.94]	[0.59–3.27]
Ashanti	0.68	3.15	1.02	1.50
	[0.27–1.71]	[0.83–11.99]	[0.55–1.91]	[0.60–3.76]
Brong Ahafo	0.53	0.65	1.11	0.93
	[0.19–1.45]	[0.11–4.03]	[0.61–2.03]	[0.31–2.81]
Northern	0.20	0.26	1.34	0.45
	[0.04–1.09]	[0.03–1.94]	[0.63–2.86]	[0.07–2.77]
Upper East	1.28	0.57	1.84	0.08^*^
	[0.42–3.88]	[0.07–5.04]	[0.89–3.80]	**[0.01**–**0.59]**
Upper West	0.37	0.00^***^	1.60	0.59
	[0.10–1.42]	**[0.00**–**0.00]**	[0.78–3.27]	[0.13–2.60]
*Place of residence*				
Rural	1	1	1	1
Urban	0.77	0.87	0.98	1.15
	[0.39–1.52]	[0.22–3.40]	[0.66–1.45]	[0.63–2.10]
*F*-statistic for goodness of fit (*p* value)	159 (0.00)		5.26 (0.00)	
Generalized Hausman test for IIA				
F-statistic (*p* value)	0.42 (0.996)	0.55 (0.972)	0.69 (0.888)	1.34 (0.117)

RRR: relative-risk ratio, results from multinomial logistic regression (reference group = short-acting contraceptive method users), survey design (sampling weights and clustering) applied; test for independence of irrelevant alternatives (IIA) implemented using *suest* Stata command to incorporate the survey design (H_o_: *difference in coefficients not systematic*), the overall statistical significance of the respective models (*F*-statistic for the goodness of fit) depicts the robustness of the models in explaining the determinants of modern contraceptive choice among Ghanaian men and women, bold indicate statistically significant values at 95% confidence interval (CI), *p < 0.05, **p < 0.01, ***p < 0.001

Marital status emerged as the single most crucial factor that influenced the choice of LARC over SAC among both men and women. Monogamous and polygamous individuals who used modern methods of contraception were more likely to choose the LARC over SAC than the unmarried. In addition, age, HIV testing status, wealth status, and educational attainment affected a woman’s choice of LARC over SAC.

Age had a nonlinear (inverted U-shaped) relationship with the probability of choosing the LARC over the SAC among women (age: RRR = 1.2, *p* < 0.05; age squared: RRR = 0.98, *p* < 0.05). Women who ever tested for HIV were more likely to use the LARC over SAC than those who never tested (RRR = 1.6, *p* < 0.01). In addition, compared to women in the highest wealth quintile (richest), those in the second (poorer: RRR = 2.2, *p* < 0.05) and third (middle: RRR = 1.9, *p* < 0.05) quintiles were more likely to opt for the LARC over SAC. Similarly, women with no formal education (RRR = 2.7, *p* < 0.05) and those with basic education (RRR = 2.5, *p* < 0.05) had a higher likelihood of choosing the LARC over SAC than women with tertiary education. Altogether, these findings suggest that the probability that a Ghanaian woman who uses modern methods of contraception chooses the LARC over SAC is higher for those with lower socioeconomic characteristics. A test for the overall effect of wealth quintile and education using the *test* Stata command supports these results. The test is statistically significant for the female sample estimation only.

Religion appears to be the most critical determinant of choosing the permanent/other method over SAC. The variable is statistically significant among both women and men. Specifically, compared to Catholics, other Christian men (excluding Charismatics) were more likely to opt for the permanent/other method over SAC (RRRR = 11.0, *p* < 0.05). In addition, Muslim men had a higher likelihood of choosing the permanent/other method over SAC than Catholics (RRR = 11.9, *p* < 0.05). In contrast, Muslim women were less likely to opt for the permanent/other method than SAC (RRR = 0.25, *p* < 0.05). Finally, the comparison between Catholic and nonreligious men was more significant than the other categories (RRR = 42.7, *p* < 0.01).

Moreover, certain determinants of choosing the permanent/other methods (marital status and administrative region) were gender specific. Men in monogamous unions were more likely to opt for the permanent/other methods than their unmarried counterparts (RRR = 3.8, *p* < 0.05).

## Discussion

Use of modern contraception is key to fertility control and population management. The study reveals interesting findings that are worth highlighting. We observe an inverted U-shaped relationship between age and modern contraception uptake for both genders. Specifically, while age increased the odds of using modern methods of contraception by 1.3 times, age squared reduced the odds by 0.96 for both genders. The plausible explanation for this relationship is that younger women and men who desire to avoid unwanted pregnancies, delay childbirth, or avoid STDs have a higher propensity to use modern methods of contraception. However, as they grow older, their tendency to use contraception declines due to reduced sexual activity. Women who have reached menopause tend to minimise the use of contraception as there is no fear of unwanted pregnancy.

Moreover, women in monogamous unions compared to the unmarried were 1.4 times more likely to use modern methods of contraception. This finding might be attributable to the desire to attain desired fertility without compromising birth spacing, which improves child and maternal health. The importance of marital status and age in explaining the uptake of modern methods of contraception is underscored in the literature [7, 13, 16].

HIV testing status emerged as a significant predictor of modern contraceptive usage among both rural and urban women. Urban women who tested for their HIV status were approximately 1.5 times (*p* < 0.01) more likely to use modern methods of contraception. This finding compares favourably with their rural counterparts, who were about 1.4 times more likely to use modern methods of contraception. The correlation with HIV testing status was consistently less pronounced in the total sample and the rural–urban sub-samples of men. The association between the HIV testing status of urban women and modern contraceptive use may be underpinned by the incentive to protect users from HIV infection (if not currently infected), avoid infecting others (if already infected) or may just reflect differences in access to health services. Condom use is most efficacious in preventing STDs, such as HIV and unwanted pregnancies [21]. Even for those infected, the use of a condom prevents experiencing an increased viral load during intimacy with HIV patients who refuse to use condoms.

Moreover, the data suggest that engaging in sexual intercourse before 17 years increases the propensity of modern contraceptive use. Men and women who engage in early sex, are found to have a higher tendency to use modern methods of contraception. However, the importance of this variable varies from rural to urban areas. For instance, men in rural areas who engaged in early sex were 1.77 times more likely to use modern methods of contraception (see Table D in Additional file 1). There was, however, no statistically significant association between urban residents and early sex for the men sample.

There are also notable regional variations in modern contraceptive use. Women resident in rural areas in Northern Ghana were 0.48 times (*p* < 0.01) less likely to use modern methods of contraception. Religious and cultural norms in the Northern Region of Ghana may explain the low probability of uptake of modern methods of contraception among women in rural northern Ghana. Nyarko in a more recent study affirmed the spatial variations in modern contraceptive use and concluded that the regional disparities in contraceptive use favour the southern regions [7]. The low uptake of modern contraception among women in the northern part of Ghana partly reflects their desire for more children.

Regarding the choice of modern methods of contraception, we find that marital status is an important predictor for both men and women. More specifically, men in monogamous unions were more likely to choose LARC and permanent/other modern contraception methods over SAC than their unmarried counterparts. Similarly, women in monogamous marriages were 1.86 times more likely to choose LARC over SAC. However, the preference for permanent/other modern methods of contraception among women is less pronounced given the lack of statistically significant association between the variables. Both men and women in polygamous unions were more likely to choose LARC over SAC. Men in polygamous marriages were 62.13 times more likely to choose LARC over SAC. At the same time, their female counterparts were 1.86 times more likely to choose LARC over SAC. Marital status, especially for men in polygamous unions, proved to be the most important predictor of choice of modern methods of contraception (RRR = 62.13, *p* < 0.001). Though polygamous men may desire larger family sizes, they may opt for LARC over SAC to reduce unwanted pregnancies with their multiple wives. It is also probable that a man may have multiple wives but might desire a relatively smaller family size, hence choosing LARC over SAC. Better still, men, being heads of their families, take the initiative in deciding on optimal family size by choosing LARC over SAC regardless of the contraception decision of their partners. For the women who chose LARC over SAC, better child spacing and desired fertility might have influenced the preference. The importance of demographic attributes, such as marital status in determining the choice of LARC over SAC is further amplified in the existing literature [22].

It is also apparent from the results that HIV testing status is an important correlate of the choice of LARC over SAC among women. The result suggests that women who know their status as HIV negative will discard using SAC, particularly condoms, and opt for LARC for family planning purposes.

Interestingly, women in the poorer and middle wealth gradients preferred LARC to SAC. Their preference for LARC has an advantage over SAC due to the recurrent expenditures on condoms and pills for contraception. Contraception failure is more associated with SAC use than the LARC and the permanent methods, since non-adherence to prescriptions could undermine the efficacy of SAC. For these reasons, some women opt for LARC over SAC as a more reliable method of modern contraception.

Moreover, relative to SAC, LARC is a preferred method of modern contraception for women with lower socioeconomic status. It is revealed that women with no formal education and those with basic education were 2.72 and 2.53 times more likely to use LARC over SAC. For these women, the fear of contraception failure and inadequate financial resources to purchase short-term contraceptives might explain their preference for LARC. In addition, it is important to note that basic education is useful in understanding the importance and efficacy of LARC as a modern method of contraception. The literature espoused the role of at least basic education in promoting modern contraception methods [22, 23].

Religion proved to be one of the most important correlates of the choice of LARC over SAC among women. Men with no religious association or inhibition were 42.74 times more likely to choose permanent/other modern methods of contraception relative to SAC. Similarly, other Christians were 10.96 times more likely to use permanent/other methods than SAC. Some religious institutions sometimes deter their adherents from using contraception. For instance, the stance of the Catholic Church on the use of modern methods of contraception prevents most of its members from using it. Some Christian faiths also propagate the preference for natural or traditional contraceptive methods over modern contraception. It is, therefore, explicable that men who are not associated with any religious beliefs have a higher propensity to use permanent/other modern methods of contraception.

## Conclusions

This paper has shed light on modern contraceptive use and choice among men and women in Ghana using the most recent and nationally representative data, GDHS, 2014. It is evident from the study that knowledge of contraceptives does not necessarily translate into usage, the reason for the comparatively low uptake of modern methods of contraception in Ghana. In addition to pooling all the modern methods of contraception together to examine the predictors of their use, we examined the socioeconomic factors that influence the choice of a specific modern contraceptive method (SAC, LARC and permanent).

It is apparent from the study that different socioeconomic factors affect the use and choice of modern contraceptive methods among men and women in Ghana. At the bivariate level, HIV testing status, early sex, wealth status and religion significantly and consistently explained modern contraceptive uptake among men and women in Ghana. At the multivariate level, marital status proved to be the most significant covariate for modern contraceptive use for both men and women. Moreover, HIV testing status and early sex were significant predictors of modern contraceptive usage in both rural and urban areas.

Regarding the choice of a specific modern method of contraception, younger women were more likely to choose LARC over SAC than older women were. In addition, women with no formal education or basic education were more likely to use LARC. It is also worth noting that relatively poorer women preferred LARC over SAC. We further observed significant regional variations in the choice of modern contraceptive methods, especially among women. An important revelation about this study is the role polygamous union, HIV testing status, wealth status, education and religious affiliation play in predicting the choice of modern methods of contraception among Ghanaian men and women.

This study indicates that access to medical insurance has no relationship with the choice of modern methods of contraception. On the contrary, we find that rural women with insurance were less likely to use modern methods of contraception (OR = 0.75, *p* < 0.01). These findings raise concerns about Ghana’s National Health Insurance Scheme (NHIS) possibly encouraging rural women to have more children. The scheme, which provided insurance cover to more than 90% of the health insured in 2012/13 [24], offers registration, premium, and renewal fees exemptions for pregnant women. Prenatal and postnatal care services are also covered under the NHIS. Interestingly, contraceptives are not covered by the scheme. This practice provides incentives for more childbirth, given the generous NHIS package for prenatal and postnatal care, including fully covered caesarean delivery. Due to unobserved confounders potentially biasing our estimates, future studies could establish the causal effect of Ghana’s NHIS on fertility or modern contraceptive use by rural women.

Despite its limitations, the paper contributes significantly to literature and policy. It provides insights into the use and determinants of modern contraceptives based on the short-acting contraceptive (SAC), long-acting contraceptive (LARC) and permanent contraceptives classifications. The paper makes a compelling case for the inclusion of contraceptives on Ghana’s National Health Insurance scheme to ensure optimal population management. The lack of statistical significance between modern contraceptive use and health insurance status is because NHIS does not currently cover contraceptives. The paper thus makes a case for sustained public health education on the health benefits of modern contraceptive use among the adult population.

## Supplementary Information

**Additional file 1. Table A.** Description and measurements of variables used in the estimations. **Table B.** Bivariate Analyses of Socioeconomics Characteristics by Modern Contraceptive Use. **Table C.** Bivariate Analyses of Socioeconomics Characteristics by Choice of Modern Method of Contraception. **Table D.** Determinants of modern contraceptive use among men. **Table E.** Determinants of modern contraceptive use among women. **Table F.** Determinants of modern contraceptive choice.

## Data Availability

The data analysed can be retrieved from the official website of the Ghana Statistical Service (https://www.statsghana.gov.gh/gssdatadownloadspage.php).

## Abbreviations

GDHS: Ghana Demographic and Health Survey
OR: Odds ratio
RRR: Relative-risk ratio
LARC: Long-acting reversible contraceptives
HIV: Human immunodeficiency virus
AIDS: Acquired immunodeficiency syndrome
IUDs: Intrauterine devices
SAC: Short-acting contraceptives
IIA: Independence of irrelevant alternatives
MNL: Multinomial logit
GSS: Ghana Statistical Service
GHS: Ghana Health Service
ICF: Inner City Fund
EAs: Enumeration areas
STDs: Sexually transmitted diseases
NHIS: National Health Insurance Scheme

## Footnotes

1 The 26% represents the average based on three surveys: (a) Ghana 2017 Performance Monitoring and Accountability 2020 Survey (27.6%), (b) Ghana 2017 Maternal Health Survey (25.0%) and (c) Ghana 2017–2018 Multiple Indicator Cluster Survey (24.3%).
