# Supplementary material for: Socioeconomic determinants of use and choice of modern contraceptive methods in Ghana
Source: Trop Med Health. 2022 May 17;50:33. doi: 10.1186/s41182-022-00424-5 (PMC9116020; doi:10.1186/s41182-022-00424-5)
Supplement: Supplementary file 1 — Additional file 1. Table A. Description and measurements of variables used in the estimations. Table B. Bivariate Analyses of Socioeconomics Characteristics by Modern Contraceptive Use. Table C. Bivariate Analyses of Socioeconomics Characteristics by Choice of Modern Method of Contraception. Table D. Determinants of modern contraceptive use among men. Table E. Determinants of modern contraceptive use among women. Table F. Determinants of modern contraceptive choice. [file 41182_2022_424_MOESM1_ESM.docx]

**Additional file 1**

**Table A. Description and measurements of variables used in the estimations**

| **Variables** | **Description and Measurement** |
| --- | --- |
|  |  |
| Modern contraceptive use | Current use of modern contraceptive methods: 1=yes, otherwise 0 |
| Modern contraceptive choice | Choice of modern method of contraception: 1=short-acting contraceptives (jelly/foam, diaphragm, pill, or condom), 2=long-acting reversible contraceptives (implants, intrauterine device, or injection), 3=permanent/other method (sterilization or others) |
| Age | Respondent’s age: in years |
| Age squared | Age squared |
| Health insured | Health insurance status: 1=insured, 0=uninsured |
| Marital status | Marital status: 0=unmarried; 1=in monogamous union; 2=in polygamous union |
| HIV tested | HIV testing status: 1=ever tested, otherwise 0 |
| Early sex | Age at first sex (early sex): 1=below 17 years, 0=17 years plus |
| Urban | Place of residence: 1=urban, 0=rural |
| Wealth quintile | Wealth status: 1=poorest, 2=poorer, 3=average, 4=rich, 5=richest^+^ |
| Education | Educational attainment: 0=no formal education, 1=basic education, 2=secondary education, 3=tertiary education ^+^ |
| Religion | Religious affiliation: 0=no religion^+^, 1=Catholic, 2=Charismatic, 3=other Christianity, 4=Islam, 5=Traditional |
| Region | Administrative region: 1=Western, 2=Central, 3=Greater Accra^+^, 4= Volta, 5= Eastern, 6=Ashanti, 7=Brong Ahafo, 8=Northern, 9=Upper East, 10=Upper West |

*Notes:* (+) represents the reference category.

**Table B. Bivariate Analyses of Socioeconomics Characteristics by Modern Contraceptive Use**

| Variable | Men | | | | | | | | | |  | Woman | | | | | | | | | |
| --- | --- | --- | --- | --- | --- | --- | --- | --- | --- | --- | --- | --- | --- | --- | --- | --- | --- | --- | --- | --- | --- |
|  | Total(*N*=4,385) | |  | Nonuser (*N*=3,495) | |  | User (*N*=890) | |  |  |  | Total (*N*=9,353) | |  | Nonuser (*N*=7,628) | |  | User(*N*=1,725) | |  |  |
|  | Mean | SD |  | Mean | SD |  | Mean | SD |  | *P*-value |  | Mean | SD |  | Mean | SD |  | Mean | SD |  | *P*-value |
| Age | 32 | 12 |  | 32 | 13 |  | 33 | 11 |  | 0.27 |  | 30 | 10 |  | 30 | 10 |  | 31 | 8 |  | 0.00 |
| Health insured | 0.49 | 0.50 |  | 0.49 | 0.50 |  | 0.49 | 0.50 |  | 0.92 |  | 0.63 | 0.48 |  | 0.63 | 0.48 |  | 0.61 | 0.49 |  | 0.30 |
| HIV tested | 0.23 | 0.42 |  | 0.21 | 0.41 |  | 0.30 | 0.46 |  | 0.00 |  | 0.48 | 0.50 |  | 0.46 | 0.50 |  | 0.61 | 0.49 |  | 0.00 |
| Engaged in early sex | 0.18 | 0.39 |  | 0.17 | 0.38 |  | 0.23 | 0.42 |  | 0.01 |  | 0.32 | 0.47 |  | 0.30 | 0.46 |  | 0.42 | 0.49 |  | 0.00 |
| Urban | 0.53 | 0.50 |  | 0.53 | 0.50 |  | 0.53 | 0.50 |  | 0.85 |  | 0.54 | 0.50 |  | 0.55 | 0.50 |  | 0.47 | 0.50 |  | 0.00 |
| *Marital status* |  |  |  |  |  |  |  |  |  |  |  |  |  |  |  |  |  |  |  |  |  |
| Unmarried | 0.48 | 0.50 |  | 0.47 | 0.50 |  | 0.50 | 0.50 |  | 0.42 |  | 0.44 | 0.50 |  | 0.46 | 0.50 |  | 0.31 | 0.46 |  | 0.00 |
| In monogamous union | 0.48 | 0.50 |  | 0.48 | 0.50 |  | 0.48 | 0.50 |  | 0.80 |  | 0.48 | 0.50 |  | 0.45 | 0.50 |  | 0.60 | 0.49 |  | 0.00 |
| In polygamous union | 0.04 | 0.20 |  | 0.04 | 0.20 |  | 0.03 | 0.16 |  | 0.01 |  | 0.09 | 0.29 |  | 0.09 | 0.29 |  | 0.09 | 0.28 |  | 0.83 |
| *Wealth quintile* | | | | | | | | | | | | | | | | | | | | | |
| Poorest | 0.17 | 0.37 |  | 0.18 | 0.39 |  | 0.12 | 0.32 |  | 0.00 |  | 0.16 | 0.37 |  | 0.16 | 0.37 |  | 0.16 | 0.37 |  | 0.73 |
| Poorer | 0.18 | 0.38 |  | 0.18 | 0.38 |  | 0.16 | 0.37 |  | 0.43 |  | 0.18 | 0.38 |  | 0.17 | 0.38 |  | 0.20 | 0.40 |  | 0.06 |
| Middle | 0.19 | 0.39 |  | 0.18 | 0.39 |  | 0.22 | 0.41 |  | 0.08 |  | 0.21 | 0.41 |  | 0.20 | 0.40 |  | 0.24 | 0.43 |  | 0.00 |
| Richer | 0.22 | 0.42 |  | 0.22 | 0.42 |  | 0.22 | 0.41 |  | 0.90 |  | 0.23 | 0.42 |  | 0.23 | 0.42 |  | 0.20 | 0.40 |  | 0.06 |
| Richest | 0.25 | 0.43 |  | 0.24 | 0.42 |  | 0.29 | 0.45 |  | 0.04 |  | 0.23 | 0.42 |  | 0.24 | 0.43 |  | 0.20 | 0.40 |  | 0.03 |
| *Educational attainment* | | | | | | | | | | | | | | | | | | | | | |
| None | 0.11 | 0.31 |  | 0.12 | 0.32 |  | 0.06 | 0.24 |  | 0.00 |  | 0.19 | 0.39 |  | 0.19 | 0.40 |  | 0.18 | 0.38 |  | 0.30 |
| Basic | 0.13 | 0.34 |  | 0.14 | 0.34 |  | 0.13 | 0.33 |  | 0.48 |  | 0.18 | 0.38 |  | 0.17 | 0.38 |  | 0.21 | 0.41 |  | 0.01 |
| Secondary | 0.64 | 0.48 |  | 0.65 | 0.48 |  | 0.63 | 0.48 |  | 0.47 |  | 0.57 | 0.50 |  | 0.57 | 0.50 |  | 0.55 | 0.50 |  | 0.15 |
| Tertiary | 0.12 | 0.32 |  | 0.10 | 0.30 |  | 0.18 | 0.39 |  | 0.00 |  | 0.06 | 0.24 |  | 0.06 | 0.24 |  | 0.06 | 0.25 |  | 0.90 |
| *Religious affiliation* |  |  |  |  |  |  |  |  |  |  |  |  |  |  |  |  |  |  |  |  |  |
| Catholic | 0.11 | 0.31 |  | 0.10 | 0.30 |  | 0.13 | 0.34 |  | 0.09 |  | 0.10 | 0.30 |  | 0.10 | 0.30 |  | 0.11 | 0.31 |  | 0.34 |
| Charismatic | 0.31 | 0.46 |  | 0.30 | 0.46 |  | 0.31 | 0.46 |  | 0.87 |  | 0.41 | 0.49 |  | 0.41 | 0.49 |  | 0.43 | 0.50 |  | 0.33 |
| Other Christian | 0.32 | 0.46 |  | 0.31 | 0.46 |  | 0.34 | 0.47 |  | 0.19 |  | 0.29 | 0.45 |  | 0.29 | 0.45 |  | 0.31 | 0.46 |  | 0.28 |
| Islam | 0.18 | 0.38 |  | 0.18 | 0.39 |  | 0.14 | 0.35 |  | 0.01 |  | 0.15 | 0.36 |  | 0.16 | 0.37 |  | 0.12 | 0.32 |  | 0.00 |
| Traditional | 0.04 | 0.19 |  | 0.04 | 0.20 |  | 0.02 | 0.15 |  | 0.02 |  | 0.02 | 0.14 |  | 0.02 | 0.15 |  | 0.01 | 0.11 |  | 0.03 |
| None | 0.06 | 0.24 |  | 0.06 | 0.24 |  | 0.06 | 0.24 |  | 0.92 |  | 0.03 | 0.16 |  | 0.03 | 0.16 |  | 0.03 | 0.18 |  | 0.19 |
| *Administrative region* | | | | | | | | | | | | | | | | | | | | | |
| Western | 0.12 | 0.32 |  | 0.12 | 0.32 |  | 0.11 | 0.31 |  | 0.58 |  | 0.11 | 0.31 |  | 0.11 | 0.31 |  | 0.12 | 0.33 |  | 0.25 |
| Central | 0.10 | 0.29 |  | 0.08 | 0.27 |  | 0.17 | 0.37 |  | 0.01 |  | 0.10 | 0.30 |  | 0.09 | 0.29 |  | 0.13 | 0.34 |  | 0.06 |
| Greater Accra | 0.22 | 0.41 |  | 0.21 | 0.41 |  | 0.26 | 0.44 |  | 0.07 |  | 0.20 | 0.40 |  | 0.21 | 0.40 |  | 0.17 | 0.38 |  | 0.07 |
| Volta | 0.07 | 0.26 |  | 0.08 | 0.27 |  | 0.05 | 0.22 |  | 0.02 |  | 0.08 | 0.27 |  | 0.07 | 0.26 |  | 0.10 | 0.30 |  | 0.04 |
| Eastern | 0.10 | 0.30 |  | 0.10 | 0.30 |  | 0.09 | 0.28 |  | 0.29 |  | 0.09 | 0.29 |  | 0.09 | 0.29 |  | 0.09 | 0.29 |  | 0.92 |
| Ashanti | 0.18 | 0.39 |  | 0.19 | 0.39 |  | 0.15 | 0.36 |  | 0.06 |  | 0.19 | 0.39 |  | 0.20 | 0.40 |  | 0.16 | 0.37 |  | 0.10 |
| Brong Ahafo | 0.08 | 0.27 |  | 0.08 | 0.27 |  | 0.08 | 0.27 |  | 0.86 |  | 0.08 | 0.28 |  | 0.08 | 0.27 |  | 0.11 | 0.31 |  | 0.01 |
| Northern | 0.08 | 0.27 |  | 0.09 | 0.28 |  | 0.04 | 0.20 |  | 0.00 |  | 0.08 | 0.28 |  | 0.09 | 0.29 |  | 0.04 | 0.20 |  | 0.00 |
| Upper East | 0.04 | 0.19 |  | 0.04 | 0.19 |  | 0.04 | 0.20 |  | 0.54 |  | 0.04 | 0.19 |  | 0.04 | 0.19 |  | 0.04 | 0.19 |  | 0.95 |
| Upper West | 0.02 | 0.15 |  | 0.02 | 0.15 |  | 0.02 | 0.14 |  | 0.32 |  | 0.02 | 0.15 |  | 0.02 | 0.15 |  | 0.02 | 0.16 |  | 0.52 |

*Notes:* Sampling weights applied to make estimates nationally representative; means, standard deviations, and *p*-values of adjusted Wald test for association reported (*H_o_*: *no relationship*).

**Table C. Bivariate Analyses of Socioeconomics Characteristics by Choice of Modern Method of Contraception**

| Variables | Men | | | | | | | | | |  | Women | | | | | | | | | |
| --- | --- | --- | --- | --- | --- | --- | --- | --- | --- | --- | --- | --- | --- | --- | --- | --- | --- | --- | --- | --- | --- |
|  | LARC (*N*=229) | |  | SAC (*N*=624) | |  | Permanent (*N*=37) | |  |  |  | LARC(*N=*1,026) | |  | SAC (*N*=568) | |  | Permanent (N=131) | |  |  |
|  | Mean | SD |  | Mean | SD |  | Mean | SD |  | P-value |  | Mean | SD |  | Mean | SD |  | Mean | SD |  | P-value |
| Age | 38 | 9 |  | 31 | 10 |  | 40 | 12 |  | 0.00 |  | 31 | 8 |  | 29 | 8 |  | 38 | 8 |  | 0.00 |
| Health insured | 0.52 | 0.50 |  | 0.48 | 0.50 |  | 0.56 | 0.50 |  | 0.50 |  | 0.63 | 0.48 |  | 0.58 | 0.50 |  | 0.64 | 0.48 |  | 0.25 |
| HIV tested | 0.32 | 0.47 |  | 0.29 | 0.45 |  | 0.38 | 0.49 |  | 0.56 |  | 0.64 | 0.48 |  | 0.55 | 0.50 |  | 0.60 | 0.49 |  | 0.01 |
| Engaged in early sex | 0.19 | 0.39 |  | 0.24 | 0.43 |  | 0.22 | 0.42 |  | 0.45 |  | 0.44 | 0.50 |  | 0.40 | 0.49 |  | 0.40 | 0.49 |  | 0.62 |
| Urban | 0.44 | 0.50 |  | 0.57 | 0.50 |  | 0.47 | 0.51 |  | 0.08 |  | 0.42 | 0.49 |  | 0.54 | 0.50 |  | 0.56 | 0.50 |  | 0.01 |
| *Marital status* |  |  |  |  |  |  |  |  |  |  |  |  |  |  |  |  |  |  |  |  |  |
| Unmarried | 0.07 | 0.25 |  | 0.67 | 0.47 |  | 0.25 | 0.44 |  | 0.00 |  | 0.24 | 0.43 |  | 0.44 | 0.50 |  | 0.27 | 0.45 |  | 0.00 |
| In monogamous union | 0.86 | 0.35 |  | 0.31 | 0.47 |  | 0.75 | 0.44 |  | 0.00 |  | 0.66 | 0.48 |  | 0.50 | 0.50 |  | 0.63 | 0.49 |  | 0.00 |
| In polygamous union | 0.07 | 0.26 |  | 0.01 | 0.11 |  | 0.00 | 0.00 |  | 0.00 |  | 0.10 | 0.31 |  | 0.06 | 0.23 |  | 0.10 | 0.30 |  | 0.01 |
| *Wealth quintile* |  |  |  |  |  |  |  |  |  |  |  |  |  |  |  |  |  |  |  |  |  |
| Poorest | 0.15 | 0.36 |  | 0.11 | 0.31 |  | 0.06 | 0.23 |  | 0.06 |  | 0.19 | 0.40 |  | 0.12 | 0.33 |  | 0.07 | 0.26 |  | 0.00 |
| Poorer | 0.21 | 0.41 |  | 0.15 | 0.35 |  | 0.16 | 0.37 |  | 0.16 |  | 0.22 | 0.41 |  | 0.16 | 0.37 |  | 0.21 | 0.41 |  | 0.01 |
| Middle | 0.23 | 0.42 |  | 0.21 | 0.40 |  | 0.30 | 0.46 |  | 0.44 |  | 0.25 | 0.43 |  | 0.23 | 0.42 |  | 0.20 | 0.41 |  | 0.52 |
| Richer | 0.18 | 0.39 |  | 0.24 | 0.43 |  | 0.18 | 0.39 |  | 0.43 |  | 0.19 | 0.39 |  | 0.24 | 0.43 |  | 0.18 | 0.38 |  | 0.16 |
| Richest | 0.23 | 0.42 |  | 0.31 | 0.46 |  | 0.31 | 0.47 |  | 0.41 |  | 0.15 | 0.36 |  | 0.25 | 0.44 |  | 0.34 | 0.48 |  | 0.01 |
| *Educational attainment* |  |  |  |  |  |  |  |  |  |  |  |  |  |  |  |  |  |  |  |  |  |
| None | 0.12 | 0.33 |  | 0.04 | 0.20 |  | 0.04 | 0.21 |  | 0.00 |  | 0.21 | 0.41 |  | 0.12 | 0.32 |  | 0.20 | 0.40 |  | 0.00 |
| Basic | 0.16 | 0.37 |  | 0.12 | 0.32 |  | 0.05 | 0.21 |  | 0.03 |  | 0.24 | 0.43 |  | 0.16 | 0.37 |  | 0.20 | 0.40 |  | 0.00 |
| Secondary | 0.54 | 0.50 |  | 0.66 | 0.48 |  | 0.70 | 0.46 |  | 0.03 |  | 0.50 | 0.50 |  | 0.63 | 0.49 |  | 0.55 | 0.50 |  | 0.00 |
| Tertiary | 0.18 | 0.38 |  | 0.18 | 0.39 |  | 0.21 | 0.41 |  | 0.94 |  | 0.05 | 0.21 |  | 0.10 | 0.30 |  | 0.05 | 0.22 |  | 0.07 |
| *Religious affiliation* |  |  |  |  |  |  |  |  |  |  |  |  |  |  |  |  |  |  |  |  |  |
| Catholic | 0.13 | 0.33 |  | 0.14 | 0.35 |  | 0.03 | 0.18 |  | 0.00 |  | 0.11 | 0.32 |  | 0.11 | 0.32 |  | 0.06 | 0.24 |  | 0.17 |
| Charismatic | 0.31 | 0.46 |  | 0.31 | 0.46 |  | 0.34 | 0.48 |  | 0.92 |  | 0.44 | 0.50 |  | 0.39 | 0.49 |  | 0.48 | 0.50 |  | 0.14 |
| Other Christian | 0.34 | 0.48 |  | 0.32 | 0.47 |  | 0.51 | 0.51 |  | 0.18 |  | 0.29 | 0.45 |  | 0.34 | 0.47 |  | 0.28 | 0.45 |  | 0.16 |
| Islam | 0.13 | 0.34 |  | 0.15 | 0.36 |  | 0.08 | 0.27 |  | 0.28 |  | 0.11 | 0.32 |  | 0.12 | 0.33 |  | 0.10 | 0.31 |  | 0.82 |
| Traditional | 0.03 | 0.18 |  | 0.02 | 0.14 |  | 0.02 | 0.15 |  | 0.50 |  | 0.01 | 0.11 |  | 0.01 | 0.10 |  | 0.01 | 0.12 |  | 0.95 |
| None | 0.06 | 0.24 |  | 0.06 | 0.24 |  | 0.01 | 0.11 |  | 0.00 |  | 0.03 | 0.18 |  | 0.02 | 0.15 |  | 0.07 | 0.25 |  | 0.29 |
| *Administrative region* |  |  |  |  |  |  |  |  |  |  |  |  |  |  |  |  |  |  |  |  |  |
| Western | 0.05 | 0.23 |  | 0.13 | 0.33 |  | 0.13 | 0.34 |  | 0.01 |  | 0.11 | 0.32 |  | 0.15 | 0.35 |  | 0.12 | 0.33 |  | 0.45 |
| Central | 0.22 | 0.41 |  | 0.15 | 0.36 |  | 0.15 | 0.36 |  | 0.38 |  | 0.12 | 0.32 |  | 0.12 | 0.33 |  | 0.24 | 0.43 |  | 0.18 |
| Greater Accra | 0.27 | 0.44 |  | 0.26 | 0.44 |  | 0.20 | 0.40 |  | 0.70 |  | 0.15 | 0.36 |  | 0.20 | 0.40 |  | 0.16 | 0.37 |  | 0.29 |
| Volta | 0.05 | 0.22 |  | 0.06 | 0.23 |  | 0.02 | 0.14 |  | 0.28 |  | 0.10 | 0.30 |  | 0.12 | 0.33 |  | 0.04 | 0.19 |  | 0.00 |
| Eastern | 0.05 | 0.22 |  | 0.10 | 0.30 |  | 0.11 | 0.32 |  | 0.15 |  | 0.10 | 0.30 |  | 0.08 | 0.27 |  | 0.12 | 0.32 |  | 0.29 |
| Ashanti | 0.14 | 0.35 |  | 0.14 | 0.35 |  | 0.33 | 0.48 |  | 0.11 |  | 0.16 | 0.36 |  | 0.16 | 0.37 |  | 0.23 | 0.42 |  | 0.34 |
| Brong Ahafo | 0.10 | 0.30 |  | 0.07 | 0.26 |  | 0.03 | 0.19 |  | 0.15 |  | 0.12 | 0.32 |  | 0.10 | 0.30 |  | 0.07 | 0.25 |  | 0.16 |
| Northern | 0.02 | 0.15 |  | 0.05 | 0.22 |  | 0.02 | 0.13 |  | 0.10 |  | 0.05 | 0.23 |  | 0.03 | 0.18 |  | 0.01 | 0.12 |  | 0.02 |
| Upper East | 0.08 | 0.27 |  | 0.03 | 0.17 |  | 0.01 | 0.11 |  | 0.00 |  | 0.05 | 0.23 |  | 0.02 | 0.15 |  | 0.00 | 0.04 |  | 0.00 |
| Upper West | 0.02 | 0.12 |  | 0.02 | 0.15 |  | 0.00 | 0.00 |  | 0.00 |  | 0.03 | 0.18 |  | 0.02 | 0.13 |  | 0.01 | 0.10 |  | 0.01 |

*Notes:* Sampling weights applied to make estimates nationally representative; means, standard deviations, and *p*-values of adjusted Wald test for association reported (*H_o_*: *no relationship*).

**Table D. Determinants of modern contraceptive use among men**

| Variables | All |  | Urban |  | Rural |  | All (with interaction) |
| --- | --- | --- | --- | --- | --- | --- | --- |
|  | OR [95% CI] |  | OR [95% CI] |  | OR [95% CI] |  | OR [95% CI] |
| Age | 1.32^***^ |  | 1.31^***^ |  | 1.36^***^ |  | 1.32^***^ |
|  | [1.23-1.41] |  | [1.21-1.43] |  | [1.23-1.50] |  | [1.23-1.41] |
| Age squared | 0.96^***^ |  | 0.96^***^ |  | 0.96^***^ |  | 0.96^***^ |
|  | [0.96-0.97] |  | [0.95-0.97] |  | [0.95-0.97] |  | [0.96-0.97] |
| **Health insurance status** |  |  |  |  |  |  |  |
| Uninsured | 1 |  | 1 |  | 1 |  | 1 |
| Insured | 1.09 |  | 1.01 |  | 1.17 |  | 1.07 |
|  | [0.89-1.35] |  | [0.76-1.35] |  | [0.86-1.60] |  | [0.87-1.32] |
| **Marital status** |  |  |  |  |  |  |  |
| Unmarried | 1 |  | 1 |  | 1 |  | 1 |
| In monogamous union | 0.50^***^ |  | 0.42^***^ |  | 0.55^**^ |  | 0.65^**^ |
|  | [0.38-0.66] |  | [0.28-0.64] |  | [0.37-0.81] |  | [0.47-0.90] |
| In polygamous union | 0.48^**^ |  | 0.40^*^ |  | 0.48^*^ |  | 0.56 |
|  | [0.29-0.79] |  | [0.18-0.90] |  | [0.24-0.94] |  | [0.30-1.07] |
| **HIV testing status** |  |  |  |  |  |  |  |
| Not tested | 1 |  | 1 |  | 1 |  | 1 |
| Tested | 1.19 |  | 1.20 |  | 1.20 |  | 1.20 |
|  | [0.92-1.54] |  | [0.88-1.63] |  | [0.77-1.88] |  | [0.93-1.56] |
| **Engaged in early sex (age<17 years)** |  |  |  |  |  |  |  |
| No | 1 |  | 1 |  | 1 |  | 1 |
| Yes | 1.47^**^ |  | 1.22 |  | 1.77^***^ |  | 1.80^***^ |
|  | [1.13-1.91] |  | [0.81-1.85] |  | [1.27-2.48] |  | [1.30-2.51] |
| **Wealth quintile (Richest)** |  |  |  |  |  |  |  |
| Richest | 1 |  | 1 |  | 1 |  | 1 |
| Poorest | 0.81 |  | 0.48 |  | 0.38 |  | 0.79 |
|  | [0.46-1.43] |  | [0.21-1.11] |  | [0.07-1.92] |  | [0.45-1.39] |
| Poorer | 0.97 |  | 0.77 |  | 0.37 |  | 0.92 |
|  | [0.60-1.57] |  | [0.42-1.41] |  | [0.08-1.77] |  | [0.57-1.47] |
| Middle | 1.17 |  | 1.06 |  | 0.36 |  | 1.03 |
|  | [0.76-1.80] |  | [0.67-1.68] |  | [0.07-1.71] |  | [0.67-1.59] |
| Richer | 0.96 |  | 0.90 |  | 0.28 |  | 0.87 |
|  | [0.66-1.38] |  | [0.61-1.33] |  | [0.06-1.34] |  | [0.59-1.26] |
| **Educational attainment** |  |  |  |  |  |  |  |
| Tertiary | 1 |  | 1 |  | 1 |  | 1 |
| None | 0.46^**^ |  | 0.90 |  | 0.29^**^ |  | 0.26^***^ |
|  | [0.28-0.77] |  | [0.44-1.82] |  | [0.14-0.61] |  | [0.13-0.50] |
| Basic | 0.74 |  | 1.09 |  | 0.49^*^ |  | 0.42^**^ |
|  | [0.48-1.14] |  | [0.57-2.10] |  | [0.26-0.92] |  | [0.24-0.74] |
| Secondary | 0.73 |  | 0.83 |  | 0.60 |  | 0.52^**^ |
|  | [0.53-1.02] |  | [0.54-1.27] |  | [0.34-1.05] |  | [0.32-0.85] |
| **Religious affiliation** |  |  |  |  |  |  |  |
| Catholic | 1 |  | 1 |  | 1 |  | 1 |
| Charismatic | 1.25 |  | 0.95 |  | 1.48 |  | 1.23 |
|  | [0.78-2.03] |  | [0.45-2.00] |  | [0.77-2.85] |  | [0.76-2.00] |
| Other Christian | 0.96 |  | 0.70 |  | 1.30 |  | 0.95 |
|  | [0.64-1.46] |  | [0.35-1.37] |  | [0.77-2.19] |  | [0.62-1.45] |
| Islam | 1.06 |  | 0.84 |  | 1.26 |  | 1.05 |
|  | [0.69-1.65] |  | [0.44-1.63] |  | [0.69-2.31] |  | [0.67-1.64] |
| Traditional | 0.92 |  | 0.69 |  | 1.00 |  | 0.87 |
|  | [0.58-1.48] |  | [0.33-1.42] |  | [0.52-1.92] |  | [0.53-1.42] |
| None | 0.89 |  | 0.62 |  | 1.14 |  | 0.86 |
|  | [0.50-1.56] |  | [0.19-1.98] |  | [0.58-2.24] |  | [0.49-1.51] |
| **Administrative region** |  |  |  |  |  |  |  |
| Greater Accra | 1 |  | 1 |  | 1 |  | 1 |
| Western | 0.72 |  | 0.74 |  | 0.65 |  | 0.70 |
|  | [0.47-1.10] |  | [0.42-1.32] |  | [0.37-1.15] |  | [0.46-1.07] |
| Central | 1.78^**^ |  | 1.63 |  | 1.75^*^ |  | 1.70^*^ |
|  | [1.17-2.71] |  | [0.89-2.99] |  | [1.01-3.03] |  | [1.14-2.55] |
| Volta | 0.51^**^ |  | 0.77 |  | 0.36^**^ |  | 0.50^**^ |
|  | [0.32-0.84] |  | [0.40-1.48] |  | [0.18-0.72] |  | [0.31-0.82] |
| Eastern | 0.71 |  | 0.96 |  | 0.52^*^ |  | 0.71 |
|  | [0.48-1.07] |  | [0.57-1.59] |  | [0.29-0.93] |  | [0.48-1.07] |
| Ashanti | 0.63^*^ |  | 0.48^**^ |  | 0.88 |  | 0.62^*^ |
|  | [0.42-0.93] |  | [0.30-0.78] |  | [0.48-1.61] |  | [0.42-0.92] |
| Brong Ahafo | 0.88 |  | 1.07 |  | 0.68 |  | 0.87 |
|  | [0.56-1.38] |  | [0.59-1.93] |  | [0.37-1.27] |  | [0.56-1.37] |
| Northern | 0.60 |  | 0.57 |  | 0.56 |  | 0.61 |
|  | [0.33-1.08] |  | [0.28-1.16] |  | [0.25-1.28] |  | [0.34-1.10] |
| Upper East | 1.32 |  | 2.97^**^ |  | 0.85 |  | 1.40 |
|  | [0.75-2.31] |  | [1.54-5.72] |  | [0.42-1.73] |  | [0.81-2.42] |
| Upper West | 0.86 |  | 2.12^**^ |  | 0.52 |  | 0.91 |
|  | [0.51-1.43] |  | [1.27-3.57] |  | [0.25-1.05] |  | [0.55-1.52] |
| **Place of residence** |  |  |  |  |  |  |  |
| Rural | 1 |  |  |  |  |  | 1 |
| Urban | 0.77 |  |  |  |  |  | 0.60 |
|  | [0.56-1.05] |  |  |  |  |  | [0.34-1.06] |
| **Marital status#urban** |  |  |  |  |  |  |  |
| In monogamous union#urban |  |  |  |  |  |  | 0.62^*^ |
|  |  |  |  |  |  |  | [0.39-0.98] |
| In polygamous union#urban |  |  |  |  |  |  | 0.67 |
|  |  |  |  |  |  |  | [0.25-1.85] |
| **Early sex#urban** |  |  |  |  |  |  |  |
| Early sex#urban |  |  |  |  |  |  | 0.69 |
|  |  |  |  |  |  |  | [0.41-1.16] |
| **Education#urban** |  |  |  |  |  |  |  |
| None#urban |  |  |  |  |  |  | 3.67^**^ |
|  |  |  |  |  |  |  | [1.48-9.12] |
| Basic#urban |  |  |  |  |  |  | 2.89^**^ |
|  |  |  |  |  |  |  | [1.31-6.39] |
| Secondary#urban |  |  |  |  |  |  | 1.65 |
|  |  |  |  |  |  |  | [0.89-3.05] |
| Observation | 4,385 |  | 2,048 |  | 2,337 |  | 4,385 |
| *F*-statistic for goodness of fit (*p*-val.) | 5.84 (0.00) |  | 4.27 (0.00) |  | 5.59 (0.00) |  | 5.82 (0.00) |

*Notes:* (a) The overall statistical significance of the respective models (*F*-statistic for goodness of fit) depicts the robustness of the models in explaining the determinants of modern contraceptive use among Ghanaian men. (b) The joint test of the interaction terms is significant for the interaction between education and urban only.

*** p<0.001, ** p<0.01, * p<0.05.

**Table E. Determinants of modern contraceptive use among women**

|  | All |  | Urban |  | Rural |  | All (with interaction) |
| --- | --- | --- | --- | --- | --- | --- | --- |
|  | OR [95% CI] |  | OR [95% CI] |  | OR [95% CI] |  | OR [95% CI] |
| Age | 1.30*** |  | 1.28*** |  | 1.33*** |  | 1.30*** |
|  | [1.23-1.39] |  | [1.14-1.42] |  | [1.24-1.43] |  | [1.22-1.39] |
| Age squared | 0.96*** |  | 0.96*** |  | 0.96*** |  | 0.96*** |
|  | [0.95-0.97] |  | [0.95-0.98] |  | [0.95-0.97] |  | [0.95-0.97] |
| **Health insurance status** |  |  |  |  |  |  |  |
| Uninsured | 1 |  | 1 |  | 1 |  | 1 |
| Insured | 0.84* |  | 0.92 |  | 0.75** |  | 0.77** |
|  | [0.73-0.97] |  | [0.75-1.13] |  | [0.62-0.90] |  | [0.64-0.92] |
| **Marital status** |  |  |  |  |  |  |  |
| Unmarried | 1 |  | 1 |  | 1 |  | 1 |
| In monogamous union | 1.44*** |  | 1.46** |  | 1.46** |  | 1.52*** |
|  | [1.20-1.73] |  | [1.12-1.89] |  | [1.15-1.85] |  | [1.20-1.92] |
| In polygamous union | 1.24 |  | 1.14 |  | 1.29 |  | 1.38* |
|  | [0.97-1.58] |  | [0.74-1.74] |  | [0.96-1.74] |  | [1.03-1.85] |
| **HIV testing status** |  |  |  |  |  |  |  |
| Not tested | 1 |  | 1 |  | 1 |  | 1 |
| Tested | 1.42*** |  | 1.51** |  | 1.35** |  | 1.37** |
|  | [1.20-1.67] |  | [1.15-1.99] |  | [1.08-1.68] |  | [1.09-1.72] |
| **Engaged in early sex (age<17 years)** |  |  |  |  |  |  |  |
| No | 1 |  | 1 |  | 1 |  | 1 |
| Yes | 1.64*** |  | 1.72*** |  | 1.61*** |  | 1.58*** |
|  | [1.40-1.91] |  | [1.35-2.21] |  | [1.31-1.97] |  | [1.31-1.89] |
| **Wealth quintile (Richest)** |  |  |  |  |  |  |  |
| Richest | 1 |  | 1 |  | 1 |  | 1 |
| Poorest | 1.38 |  | 0.69 |  | 0.84 |  | 1.38 |
|  | [0.96-1.97] |  | [0.38-1.24] |  | [0.28-2.48] |  | [0.96-1.98] |
| Poorer | 1.20 |  | 1.06 |  | 0.65 |  | 1.20 |
|  | [0.83-1.73] |  | [0.71-1.57] |  | [0.20-2.14] |  | [0.83-1.74] |
| Middle | 1.23 |  | 1.28 |  | 0.61 |  | 1.24 |
|  | [0.94-1.60] |  | [0.98-1.67] |  | [0.21-1.76] |  | [0.95-1.62] |
| Richer | 1.01 |  | 1.07 |  | 0.42 |  | 1.01 |
|  | [0.81-1.26] |  | [0.85-1.35] |  | [0.16-1.12] |  | [0.81-1.27] |
| **Educational attainment** |  |  |  |  |  |  |  |
| Tertiary | 1 |  | 1 |  | 1 |  | 1 |
| None | 0.75 |  | 1.03 |  | 0.49 |  | 0.75 |
|  | [0.49-1.14] |  | [0.62-1.70] |  | [0.23-1.07] |  | [0.49-1.14] |
| Basic | 0.94 |  | 1.11 |  | 0.69 |  | 0.95 |
|  | [0.63-1.40] |  | [0.68-1.82] |  | [0.34-1.41] |  | [0.64-1.41] |
| Secondary | 0.91 |  | 0.97 |  | 0.74 |  | 0.92 |
|  | [0.62-1.33] |  | [0.63-1.50] |  | [0.36-1.54] |  | [0.63-1.34] |
| **Religious affiliation** |  |  |  |  |  |  |  |
| Catholic | 1 |  | 1 |  | 1 |  | 1 |
| Charismatic | 0.94 |  | 0.96 |  | 0.95 |  | 0.95 |
|  | [0.62-1.43] |  | [0.44-2.11] |  | [0.59-1.53] |  | [0.63-1.45] |
| Other Christian | 0.90 |  | 0.81 |  | 1.03 |  | 0.91 |
|  | [0.61-1.33] |  | [0.38-1.73] |  | [0.65-1.62] |  | [0.61-1.34] |
| Islam | 0.93 |  | 1.04 |  | 0.84 |  | 0.94 |
|  | [0.64-1.35] |  | [0.50-2.14] |  | [0.57-1.23] |  | [0.65-1.35] |
| Traditional | 0.77 |  | 0.70 |  | 0.86 |  | 0.79 |
|  | [0.53-1.13] |  | [0.33-1.50] |  | [0.56-1.33] |  | [0.54-1.15] |
| None | 0.54* |  | 0.95 |  | 0.48* |  | 0.53* |
|  | [0.29-1.00] |  | [0.20-4.50] |  | [0.25-0.92] |  | [0.29-0.99] |
| **Administrative region** |  |  |  |  |  |  |  |
| Greater Accra | 1 |  | 1 |  | 1 |  | 1 |
| Western | 1.23 |  | 1.36 |  | 1.20 |  | 1.22 |
|  | [0.88-1.72] |  | [0.93-1.99] |  | [0.73-1.99] |  | [0.87-1.70] |
| Central | 1.39 |  | 1.23 |  | 1.49 |  | 1.38 |
|  | [1.00-1.94] |  | [0.83-1.82] |  | [0.96-2.33] |  | [0.99-1.93] |
| Volta | 1.47* |  | 1.08 |  | 1.72* |  | 1.47* |
|  | [1.02-2.13] |  | [0.69-1.70] |  | [1.04-2.83] |  | [1.01-2.12] |
| Eastern | 1.03 |  | 1.41 |  | 0.78 |  | 1.02 |
|  | [0.74-1.43] |  | [0.97-2.05] |  | [0.50-1.23] |  | [0.74-1.42] |
| Ashanti | 0.93 |  | 0.92 |  | 1.00 |  | 0.93 |
|  | [0.68-1.29] |  | [0.64-1.32] |  | [0.59-1.69] |  | [0.67-1.28] |
| Brong Ahafo | 1.47* |  | 1.20 |  | 1.72* |  | 1.45* |
|  | [1.06-2.03] |  | [0.83-1.75] |  | [1.06-2.77] |  | [1.04-2.01] |
| Northern | 0.51** |  | 0.63 |  | 0.48** |  | 0.51** |
|  | [0.34-0.77] |  | [0.30-1.33] |  | [0.30-0.78] |  | [0.34-0.77] |
| Upper East | 1.08 |  | 2.10*** |  | 0.86 |  | 1.07 |
|  | [0.75-1.56] |  | [1.36-3.23] |  | [0.56-1.32] |  | [0.74-1.55] |
| Upper West | 1.22 |  | 1.68 |  | 1.12 |  | 1.22 |
|  | [0.82-1.82] |  | [0.97-2.89] |  | [0.68-1.85] |  | [0.82-1.81] |
| **Place of residence** |  |  |  |  |  |  |  |
| Rural | 1 |  | 1 |  | 1 |  | 1 |
| Urban | 0.83 |  | 1.00 |  | 1.00 |  | 0.72 |
|  | [0.66-1.03] |  | [1.00-1.00] |  | [1.00-1.00] |  | [0.51-1.02] |
| **Insured#urban** |  |  |  |  |  |  | 1.22 |
|  |  |  |  |  |  |  | [0.93-1.61] |
| **Marital status#urban** |  |  |  |  |  |  |  |
| In monogamous union#urban |  |  |  |  |  |  | 0.91 |
|  |  |  |  |  |  |  | [0.66-1.23] |
| In polygamous union#urban |  |  |  |  |  |  | 0.75 |
|  |  |  |  |  |  |  | [0.46-1.20] |
| **Tested#urban** |  |  |  |  |  |  | 1.09 |
|  |  |  |  |  |  |  | [0.77-1.53] |
| **Early sex#urban** |  |  |  |  |  |  | 1.10 |
|  |  |  |  |  |  |  | [0.81-1.48] |
| Observation | 9,353 |  | 4,575 |  | 4,778 |  | 9,353 |
| *F*-statistic for goodness of fit (*p*-val.) | 11.42 (0.00) |  | 5.16 (0.00) |  | 10.01 (0.00) |  | 9.73 (0.00) |

*Notes:* (a) The overall statistical significance of the respective models (*F*-statistic for goodness of fit) depicts the robustness of the models in explaining the determinants of modern contraceptive use among Ghanaian women. (b) The joint test of the interaction terms is insignificant for all the interaction terms.

*** p<0.001, ** p<0.01, * p<0.05.

**Table F. Determinants of modern contraceptive choice**

| Explanatory variables | Men | | |  | Women | | |
| --- | --- | --- | --- | --- | --- | --- | --- |
|  | LARC |  | Permanent/other |  | LARC |  | Permanent/other |
|  | RRR [95% CI] |  | RRR [95% CI] |  | RRR [95% CI] |  | RRR [95% CI] |
| Age | 1.16 |  | 0.92 |  | 1.16^*^ |  | 1.03 |
|  | [0.93-1.45] |  | [0.69-1.23] |  | [1.03-1.31] |  | [0.75-1.41] |
| Age squared | 0.98 |  | 1.02 |  | 0.98^*^ |  | 1.01 |
|  | [0.95-1.01] |  | [0.98-1.06] |  | [0.96-1.00] |  | [0.97-1.06] |
| **Health insurance status** |  |  |  |  |  |  |  |
| Uninsured | 1 |  | 1 |  | 1 |  | 1 |
| Insured | 1.01 |  | 1.04 |  | 1.20 |  | 1.75 |
|  | [0.63-1.61] |  | [0.45-2.41] |  | [0.91-1.60] |  | [0.95-3.21] |
| **Marital status** |  |  |  |  |  |  |  |
| Unmarried | 1 |  | 1 |  | 1 |  | 1 |
| In monogamous union | 21.59^***^ |  | 3.75^*^ |  | 1.86^***^ |  | 0.91 |
|  | [8.17-57.06] |  | [1.08-13.02] |  | [1.36-2.54] |  | [0.46-1.78] |
| In polygamous union | 62.13^***^ |  | 0.00^***^ |  | 2.32^**^ |  | 1.03 |
|  | [16.49-234.13] |  | [0.00-0.00] |  | [1.39-3.88] |  | [0.31-3.38] |
| **HIV testing status** |  |  |  |  |  |  |  |
| Not tested | 1 |  | 1 |  | 1 |  | 1 |
| Tested | 1.08 |  | 1.15 |  | 1.55^**^ |  | 1.45 |
|  | [0.60-1.95] |  | [0.50-2.64] |  | [1.15-2.08] |  | [0.82-2.59] |
| **Engaged in early sex (age<17 years)** | |  |  |  |  |  |  |
| No | 1 |  | 1 |  | 1 |  | 1 |
| Yes | 0.80 |  | 0.76 |  | 1.04 |  | 1.25 |
|  | [0.44-1.47] |  | [0.23-2.57] |  | [0.70-1.55] |  | [0.75-2.10] |
| **Wealth quintile (Richest)** |  |  |  |  |  |  |  |
| Richest | 1 |  | 1 |  | 1 |  | 1 |
| Poorest | 1.28 |  | 0.60 |  | 1.96 |  | 0.40 |
|  | [0.32-5.18] |  | [0.05-6.77] |  | [0.90-4.25] |  | [0.11-1.46] |
| Poorer | 2.04 |  | 0.85 |  | 2.19^*^ |  | 0.71 |
|  | [0.68-6.13] |  | [0.14-4.98] |  | [1.19-4.01] |  | [0.29-1.74] |
| Middle | 2.32 |  | 1.82 |  | 1.92^*^ |  | 0.60 |
|  | [0.79-6.80] |  | [0.35-9.61] |  | [1.15-3.20] |  | [0.28-1.28] |
| Richer | 1.41 |  | 1.00 |  | 1.29 |  | 0.50 |
|  | [0.49-4.06] |  | [0.26-3.80] |  | [0.78-2.14] |  | [0.23-1.08] |
| **Educational attainment** |  |  |  |  |  |  |  |
| Tertiary | 1 |  | 1 |  | 1 |  | 1 |
| None | 1.47 |  | 0.80 |  | 2.72^*^ |  | 3.19 |
|  | [0.49-4.39] |  | [0.12-5.27] |  | [1.20-6.14] |  | [0.99-10.29] |
| Basic | 1.04 |  | 0.35 |  | 2.53^*^ |  | 1.97 |
|  | [0.41-2.67] |  | [0.06-1.94] |  | [1.20-5.34] |  | [0.53-7.23] |
| Secondary | 0.93 |  | 0.80 |  | 1.56 |  | 1.94 |
|  | [0.43-2.05] |  | [0.29-2.23] |  | [0.73-3.32] |  | [0.77-4.91] |
| **Religious affiliation** |  |  |  |  |  |  |  |
| Catholic | 1 |  | 1 |  | 1 |  | 1 |
| Charismatic | 1.11 |  | 2.47 |  | 0.71 |  | 0.20 |
|  | [0.39-3.18] |  | [0.15-39.86] |  | [0.29-1.71] |  | [0.04-1.09] |
| Other Christian | 1.32 |  | 10.96^*^ |  | 0.94 |  | 0.40 |
|  | [0.44-3.90] |  | [1.16-103.43] |  | [0.41-2.17] |  | [0.12-1.31] |
| Islam | 1.02 |  | 11.94^*^ |  | 0.80 |  | 0.25^*^ |
|  | [0.37-2.81] |  | [1.20-118.59] |  | [0.33-1.92] |  | [0.07-0.89] |
| Traditional | 0.60 |  | 6.28 |  | 0.51 |  | 0.24 |
|  | [0.19-1.92] |  | [0.48-81.54] |  | [0.20-1.30] |  | [0.05-1.28] |
| None | 0.87 |  | 42.74^**^ |  | 0.55 |  | 0.50 |
|  | [0.21-3.51] |  | [3.20-571.40] |  | [0.16-1.86] |  | [0.08-2.90] |
| **Administrative region** |  |  |  |  |  |  |  |
| Greater Accra | 1 |  | 1 |  | 1 |  | 1 |
| Western | 0.25^*^ |  | 1.48 |  | 0.79 |  | 1.25 |
|  | [0.08-0.76] |  | [0.31-7.13] |  | [0.41-1.49] |  | [0.54-2.89] |
| Central | 0.53 |  | 0.76 |  | 0.97 |  | 2.33 |
|  | [0.19-1.43] |  | [0.13-4.66] |  | [0.53-1.77] |  | [0.95-5.69] |
| Volta | 0.43 |  | 0.35 |  | 0.63 |  | 0.34 |
|  | [0.13-1.39] |  | [0.02-6.34] |  | [0.34-1.16] |  | [0.11-1.05] |
| Eastern | 0.30 |  | 1.33 |  | 1.09 |  | 1.39 |
|  | [0.08-1.10] |  | [0.26-6.78] |  | [0.61-1.94] |  | [0.59-3.27] |
| Ashanti | 0.68 |  | 3.15 |  | 1.02 |  | 1.50 |
|  | [0.27-1.71] |  | [0.83-11.99] |  | [0.55-1.91] |  | [0.60-3.76] |
| Brong Ahafo | 0.53 |  | 0.65 |  | 1.11 |  | 0.93 |
|  | [0.19-1.45] |  | [0.11-4.03] |  | [0.61-2.03] |  | [0.31-2.81] |
| Northern | 0.20 |  | 0.26 |  | 1.34 |  | 0.45 |
|  | [0.04-1.09] |  | [0.03-1.94] |  | [0.63-2.86] |  | [0.07-2.77] |
| Upper East | 1.28 |  | 0.57 |  | 1.84 |  | 0.08^*^ |
|  | [0.42-3.88] |  | [0.07-5.04] |  | [0.89-3.80] |  | [0.01-0.59] |
| Upper West | 0.37 |  | 0.00^***^ |  | 1.60 |  | 0.59 |
|  | [0.10-1.42] |  | [0.00-0.00] |  | [0.78-3.27] |  | [0.13-2.60] |
| **Place of residence** |  |  |  |  |  |  |  |
| Rural | 1 |  | 1 |  | 1 |  | 1 |
| Urban | 0.77 |  | 0.87 |  | 0.98 |  | 1.15 |
|  | [0.39-1.52] |  | [0.22-3.40] |  | [0.66-1.45] |  | [0.63-2.10] |
| Observation | 890 | | |  | 1,725 | | |
| *F*-statistic for goodness of fit (*p*-val.) | 159 (0.00) | | |  | 5.26 (0.00) | | |
| Generalized Hausman test for IIA |  |  |  |  |  |  |  |
| F-statistic (*p*-val.) | 0.42(0.996) |  | 0.55(0.972) |  | 0.69(0.888) |  | 1.34(0.117) |

Notes: RRR = relative-risk ratio. (a) The overall statistical significance of the respective models (*F*-statistic for goodness of fit) depicts the robustness of the models in explaining the determinants of modern contraceptive choice among Ghanaian men and women. (b) Test for independence of irrelevant alternatives (IIA) were implemented using *suest* Stata command to incorporate the survey design (*H*_o_: *difference in coefficients not systematic*).

*** p<0.001, ** p<0.01, * p<0.05
